# A multi-omic analysis reveals the role of fumarate in regulating the virulence of enterohemorrhagic *Escherichia coli*

**DOI:** 10.1038/s41419-018-0423-2

**Published:** 2018-03-07

**Authors:** Cheng-Ju Kuo, Sin-Tian Wang, Chia-Mei Lin, Hao-Chieh Chiu, Cheng-Rung Huang, Der-Yen Lee, Geen-Dong Chang, Ting-Chen Chou, Jenn-Wei Chen, Chang-Shi Chen

**Affiliations:** 1 Department of Biochemistry and Molecular Biology, College of Medicine, National Cheng Kung University, Tainan, Taiwan; 2 Institute of Basic Medical Sciences, College of Medicine, National Cheng Kung University, Tainan, Taiwan; 3 Department of Clinical Laboratory Sciences and Medical Biotechnology, National Taiwan University, Taipei, Taiwan; 4 The Graduate Institute of Integrated Medicine, China Medical University, Taichung, Taiwan; 5 Graduate Institute of Biochemical Sciences, Technology Commons, Center for Systems Biology, National Taiwan University, Taipei, Taiwan; 6 Department of Microbiology and Immunology, College of Medicine, National Cheng Kung University, Tainan, Taiwan

## Abstract

The enteric pathogen enterohemorrhagic *Escherichia coli* (EHEC) is responsible for outbreaks of bloody diarrhea and hemolytic uremic syndrome (HUS) worldwide. Several molecular mechanisms have been described for the pathogenicity of EHEC; however, the role of bacterial metabolism in the virulence of EHEC during infection in vivo remains unclear. Here we show that aerobic metabolism plays an important role in the regulation of EHEC virulence in *Caenorhabditis elegans*. Our functional genomic analyses showed that disruption of the genes encoding the succinate dehydrogenase complex (Sdh) of EHEC, including the *sdhA* gene, attenuated its toxicity toward *C. elegans* animals. Sdh converts succinate to fumarate and links the tricarboxylic acid (TCA) cycle and the electron transport chain (ETC) simultaneously. Succinate accumulation and fumarate depletion in the EHEC *sdhA* mutant cells were also demonstrated to be concomitant by metabolomic analyses. Moreover, fumarate replenishment to the *sdhA* mutant significantly increased its virulence toward *C. elegans*. These results suggest that the TCA cycle, ETC, and alteration in metabolome all account for the attenuated toxicity of the *sdhA* mutant, and Sdh catabolite fumarate in particular plays a critical role in the regulation of EHEC virulence. In addition, we identified the tryptophanase (TnaA) as a downstream virulence determinant of SdhA using a label-free proteomic method. We demonstrated that expression of *tnaA* is regulated by fumarate in EHEC. Taken together, our multi-omic analyses demonstrate that *sdhA* is required for the virulence of EHEC, and aerobic metabolism plays important roles in the pathogenicity of EHEC infection in *C. elegans*. Moreover, our study highlights the potential targeting of SdhA, if druggable, as alternative preventive or therapeutic strategies by which to combat EHEC infection.

## Introduction

Enterohemorrhagic *Escherichia coli* (EHEC) is one of the Shiga-like toxin (Stx) producing pathogens, and can cause severe diarrhea, hemorrhagic colitis, and hemolytic uremic syndrome (HUS) in humans^1^. Besides the Stxs in EHEC, several other virulence determinants have been identified, including the locus of enterocyte effacement (LEE) and the F-like virulence plasmid *pO157*^2^. It has been reported EHEC can produce indoles, from tryptophan metabolism catalyzed by the tryptophanase TnaA, to paralyze and kill *C. elegans* animals^3,4^. Moreover, the secreted indoles produced by EHEC act as signal molecules to regulate LEE genes expression, and together with the *tnaA* gene are required for the formation of attaching and effacing (A/E) lesions in cultured mammalian cells^5^. Aerobic respiration has also been implicated to be required for the colonization of EHEC in the mouse intestine^6^. This report is in agreement with the notion that a zone adjacent to the GI tract mucosa, the potential in vivo microenvironment for EHEC, is aerobic caused by diffusion of oxygen from the capillary network at the tips of microvilli^7^. An in vitro study also showed that the expression profile of the LEE genes in EHEC can be regulated by the catabolite of glucose metabolism, fructose-1, 6-bisphsphate (FBP), in a catabolite repressor/activator protein Cra-dependent manner^8^. These reports together suggest that carbon and central metabolic pathways might regulate the pathogenesis of EHEC in the localized microenvironment in vivo.

In order to adapt to local microenvironments, escape host defense, and causes disease during infection within the GI tract, EHEC can sense nutrients and chemical signals produced by either host or commensal microbiota^9–12^. EHEC also responds to lipid metabolites produced by host and microbiota in the ileum and colon, such as short-chain fatty acids (SCFAs) and ethanolamine (EA), to modulate the motility, adhesion, and virulence^13–16^. Moreover, the transportation and utilization of fucose, made available through the cleavage of host glycans by commensal microbiota, modulates EHEC pathogenicity in a FusKR two component system-dependent manner^17^. All these reports support the notion that nutrient metabolism and metabolites from host and microbiota work in concert to regulate EHEC virulence in vivo.

Owing to the lack of mouse models for studying EHEC infection^18^, we and others applied the model organism *C. elegans* to study EHEC infection in vivo^3,19^. By screening an EHEC transposon mutagenesis library, we have identified potential bacterial virulence determinants involved in the pathogenicity of EHEC in *C. elegans*. Of note, we identified one transposon mutant of the *sdhA* gene and one mutant of the *sdhC* gene, these two genes encode the catalytic and ubiquinone interacting subunits respectively for the succinate dehydrogenase complex (Sdh). Sdh serves dual roles both in the TCA cycle (the sixth enzyme) and ETC (the complex II) in aerobic metabolism. However, the roles of bacterial central metabolism, including the TCA cycle and ETC, in regulating the virulence of EHEC during infection are still largely unknown. Herein, we further characterized how loss-of-function of the *sdh* genes, particularly in the *sdhA* mutants, can influence the virulence of EHEC, and how a Sdh catabolite, i.e., fumarate, could regulate the pathogenicity of EHEC in *C. elegans*.

## Materials and methods

### Nematode strains and maintenance of *C. elegans*

The nematode strains used in this study are listed in Supplemental Table S1. Worms were handled in the laboratory as described previously and were maintained on Nematode Growth Medium (NGM) agar plate fed with the standard and non-pathogenic laboratory *E. coli* strain OP50 as a normal food source^20^.

### Bacterial strains and plasmids

The bacterial strains used in this study are listed in Supplemental Table S2. The enterohemorrhagic *E. coli* O157:H7 clinical isolates, including the EDL933 strain and the HER1266 strain, were from the Bioresource Collection and Research Center, Taiwan. All EHEC-related biohazardous wastes were disinfected and disposed according to the Biosafety Level 2 (BSL-2) regulation. All bacterial gene deletion strains were constructed by using the Lambda Red recombinase system^21,22^ described in the supplemental information section. The genotypes/mutations of all bacterial strains used in the study were confirmed by DNA sequencing. The plasmids used in this study are listed in Supplemental Table S3. The ampicillin-resistant plasmid *pFPV25.1*, which constitutively expressed green fluorescent protein (GFP)^23^, was transformed to OP50 and EDL933 strain to determine colonization status of live bacteria in the intestine of *C. elegans*. The *sdhCDAB* operon expressing plasmid, *pWF134*, was constructed by cloning the whole *sdhCDAB* operon (4769 bp) into the expression vector pQE30 (Qiagen) between the *Sph*I and *Sal*I restriction enzyme sites. The *sdhCDAB* operon was amplified by PCR from EDL933 genomic DNA with the primers 5′-ACA TGC ATG CTT AAG GTC TCC TTA GCG CC-3′ and 5′-ACG CGT CGA CGC CGC ATC CGG CAC TGG TTG-3′. The PCR amplified fragment was double digested with *Sph*I and *Sal*I restriction enzymes (NEB). The digested PCR product was extracted from agarose gels with QIAquick Gel Extraction Kit (Qiagen), and then was ligated *with Sph*I*/Sal*I-digested pQE30 vector to obtain the plasmid pWF134. Authentication of all the plasmids used in this study was reconfirmed by DNA sequencing. All the primers used in the study are list in Supplemental Table S4.

### Construction of the EHEC transposon mutagenesis library

To generate the EDL933 genome-wide transposon mutants, we used the EZ-Tn*5* < R6Kγori/KAN-2 > Tnp Transposome Kit (Epicentre). The EZ-Tn*5* < R6Kγ ori/KAN-2 > Transposon contains an R6Kγ conditional origin of replication (R6Kγ ori) and the Tn*903* kanamycin resistance gene (Kan^R^) that is functional in *E. coli*, flanked by hyperactive 19 base pair Mosaic End (ME) EZ-Tn*5* Transposase recognition sequences. The EZ-Tn*5* system has allowed random mutagenesis in many bacterial species (www.epibio.com). To obtain EDL933 mutants, a 100 μL aliquot of the EDL933 electrocompetent cells was mixed with 1 μL (25 ng/μL) transposon DNA (EZ-Tn*5* < R6Kγori/KAN-2 > Tnp Transposome Mutagenesis kit), incubated at 37 °C overnight. The Kan^R^ colonies were picked into the 96-well plates with LB broth contained 50 μg/mL kanamycin, and the entire EDL933 transposome library was stored at −80 °C.

### Screening of the EDL933 transposome library

The detailed screening method is described and illustrated in the Supplemental Information (Figure S1). In brief, the L1 stage *C. elegans glp-4(bn2)* mutant animals, with germ-line proliferation defect developed at the restrictive temperature (25 °C), were seeded on an NGM plate with *E. coli* OP50 at Day 1. At the same time, the EDL 933 transposon mutagenesis library, stored in 96-well plates, were replicated in LB broth containing 50 μg/mL Kanamycin (Kan) and put in a 37 °C incubator for 16–18 h. On the next day (Day 2), the entire library was triplicated into 96-well plates containing LB broth with 50 μg/mL Kan and cultured at 37 °C for another 16 to 18 h. At Day 3 until *glp-4 (bn2)* animals had reached to the L4 larva to young adult stage, the worms were washed by M9 buffer and added into the 96-well plates (15–20 worms/well) and incubated at 25 °C with constant agitation at 70 rpm. After 8 days, the O.D._595_ values of each well were detected by DTX 800 Multimode Detector (Beckman Coulter). The O.D._595_ value was close to 0.5 when worms were cultured with *E. coli* strain OP50 (as negative control). In contrast, the O.D._595_ value was around 1.0 when the worms were fed with EHEC wild-type EDL933 (as positive control). The hits/candidates with a decreased pathogenic phenotype toward *C. elegans* were selected with the O.D. value that was significantly lower compared to the EHEC wild-type EDL933 positive controls (*P* < 0.05).

### Identification of the mutation sites of the selected Tn5 transposition clones

The insertion site of any chosen EDL933 mutant clones was determined by the method described in the EZ-Tn*5* Transposome Kit (Epicentre). In brief, the genomic DNA from the clone of interest was prepared by EasyPure Genomic DNA Spin kit (Bioman Scientific). Then, the genomic DNA was digested by *Eco*RV that cuts near the end of the EZ-Tn*5* transposon. The resulted fragmented genomic DNA was self-ligated by T4 ligase. The ligation mixture was electroporated into the *E. coli pir + *electrocompetent cell and selected by Kan. Finally, the plasmids DNA of the rescued Kan^R^ clones were sequenced using the forward and reverse EZ-Tn*5* < R6Kγ ori/KAN-2 > Transposon-specific primers supplied in the kit system to identify the insertion site.

### EHEC killing assays

*C. elegans* plate-based killing by *E. coli* O157:H7 strains were conducted as our previous published procedures^19,24^. In brief, 30 μL of the overnight *E*. *coli* LB broth culture was spread on 5.0 cm NGM agar plates and incubated overnight at 37 °C. On the next day, after equilibrating the plates to room temperature, about 50 synchronized late L4 to young adult stage *C. elegans* animals were transferred to each plate and kept at 20 °C. Animals were transferred to fresh plates daily during the progeny production period and monitored daily for dead animals. The experiment was performed independently three times with approximately 100 worms per *E*. *coli* strain each time at 20 °C. Survival analysis was performed using GraphPad Prism 6.0 (GraphPad Software, La Jolla, CA). The Mantel–Cox log-rank test was used to assess statistical significance of difference in survival, and *P* values <0.05 were considered significant. For the succinate and fumarate treatment assays, bacteria were grown overnight at 37 °C in LB broth containing 2.5 mM succinic acid (Merck) or 2.5 mM fumaric acid (Merck) and adjusted to pH 6.6 by NaOH, and spread on NGM agar plates also containing 2.5 mM succinic acid or 2.5 mM fumaric acid and adjusted to pH 6.2 by NaOH. For the anaerobic condition assay, NGM agar plates containing bacterial lawn (100 μL per plate) were grown at BBL GasPak Anaerobic Systems at room temperature overnight. To ensure that worms were fed with bacteria from anaerobic culture continuously, worms were transferred to fresh prepared plates daily.

### Quantification of bacterial intestinal colonization

Measurement of live bacterial number colonized in the intestine of *C. elegans* was performed as our previous published procedures^19,24^. In brief, N2 worms were fed with bacteria, including OP50, EDL933, EDL933:Δ*sdhA*, or EDL933:Δ*sdhA*-*pWF134*, for 1 day at 20 °C and then transferred to the OP50 bacterial plates for another three days at 20 °C. In order to keep the GFP-expressing *pFPV25.1* plasmid, OP50-GFP, and EDL933-GFP were inoculated on the plates with ampicillin. On day 4, worms were washed out from the plate using M9 buffer treated with 25 mM levamisole and collected by centrifugation at 1000×*g* for 1 min. The infected worms were washed by M9 buffer containing 25 mM levamisole for another 10 times. The infected worms then were incubated with M9 containing 25 mM levamisole, 100 mg/mL gentamicin and 1 mg/mL ampicillin for 1–2 h at room temperature to remove the bacteria outside of the animals. These antibiotics were eliminated by washing the worms in M9 buffer with 25 mM levamisole three times. After the final wash, ten worms were picked randomly into 100 μL M9 buffer in an Eppendorf microtube, pulverized for 1 min using a sterile plastic pestle, and plated on LB agar containing ampicillin after serial dilution. The number of bacterial cells (colony number) was determined and the colony-forming unit (CFU) per worm was calculated. Student’s *t-*test was used to determine the significance of the differences (*P* < 0.05).

### Microvillar ACT-5 cellular localization

Measurement of ectopic ACT-5 expression in the intestine of *C. elegans* was performed as described^19,24^. In brief, L4 larvae of the GK454 strain, the *unc-119* mutant with *dkIs247*[*act-5p::mCherry::HA::act-5*, *unc119(+)*] transgene, were fed on bacterial lawns of OP50, EDL933, EDL933:Δ*sdhA*, or EDL933:Δ*sdhA*-*pWF134* for 4 days at 20 °C. The microvillar ACT-5 ectopic expression was determined by fluorescence microscopy.

### Metabolomic analysis

Bacteria were cultured in LB broth at 37 °C for 16–18 h with shaking at 220 rpm. Bacteria cells for, 40 mL bacterial culture was harvested by centrifugation at 10,000 rpm for 15 min at 4 °C, and the cell pellet was washed by 5 mL deionized water three times at 4 °C. The cell pellets were resuspended in 500 μL deionized water. The bacterial solution was adjusted to 10^8^–10^9^ colony-forming units per mL. The bacterial metabolite was extracted by using 100% MeOH, and was vortexed for 5 min. After centrifugation for 10 min at 14,000 rpm, 200 μL of supernatant was taken to be vacuum dried at room temperature. The dry sample was reconstituted in 100 μL of 80% MeOH and then centrifuged at 14,000 rpm for 10 min. The supernatant was collected and subjected to LC-ESI-MS analysis of positive or negative ion mode. The LC-ESI-MS system consisted of an ultra-performance liquid chromatography (UPLC) system (Ultimate 3000 RSLC, Dionex) and an electrospray ionization (ESI) source of quadrupole time-of-flight (TOF) mass spectrometer (maXis HUR-QToF system, Bruker Daltonics). The acquired data were processed by TargetAnalysis and DataAnalysis software (Bruker Daltonics) with summarized in an integrated area of signals. Found compounds were selected with the tolerance of LC peaks within 0.3 min and area higher than 1000 counts from established compound identities. The fold change of the metabolite was determined by amount compared to wild-type EHEC EDL933. Student’s *t-*test was used to determine the significance of the differences (*P* < 0.05)

### Real-time quantitative RT-PCR

Bacteria were cultivated in LB broth at 37 °C for 16–18 h with shaking at 220 rpm. The overnight bacterial cultures were diluted 1:1000 into fresh LB broth at 37 °C with shaking at 220 rpm until cells reached log-phase growth (O.D._600_ value is 0.4–0.6). Bacterial cultures were treated with RNAprotect Bacteria Reagent (Qiagen) to stabilize total RNA in bacteria, and total RNA was purified by RNeasy minikit and DNA was removed with on-column DNase digestion (Qiagen). Purified RNAs were quantified with a NanoDrop spectrophotometer, and were reverse transcribed into complementary DNA with M-MLV reverse transcriptase (Promega) using random hexamer primers. Quantitative real time-PCR was performed with 7500 Fast Real-Time PCR system (Applied Biosystems) using SYBR green PCR Master Mix (Roche). RNA polymerase subunit A, *rpoA*, was used as an internal control, and relative transcriptional expression of each gene was normalized to the level of *rpoA*. The fold-change of gene expression was determined via *ΔΔ*Ct values compared to wild-type EHEC EDL933. Student’s *t-*test was used for statistical analysis, and *P* values less than 0.05 were regarded as statistically significant. The primers used in qRT-PCR are listed in Table S4.

### Proteomic analysis

#### Sample preparation

Three bacteria strains, EDL933, EDL933:Δ*sdhA*, or EDL933:Δ*sdhA*-*pWF134*, were cultured in LB broth at 37 °C for 16–18 h with agitation at 220 rpm. The overnight bacterial cultures were diluted 1:50 into fresh LB broth and cultured to an O.D._600_ of 2.0 at 37 °C with shaking (220 rpm). Bacterial cells were collected from 5 mL of bacterial culture by centrifugation at 9000 rpm for 10 min at 4 °C, and were then washed with 10 mL 10 mM Tris-HCl, 5 mM magnesium acetate (pH 8.0), followed by centrifugation at 9000 rpm for 10 min at 4 °C. The wash procedures were repeated another four times in order to prevent the proteins from LB broth interfering with the bacterial proteomes. Three independent biological repeats were prepared for each bacterial strain. The final numbers of bacteria were between 10^8^ and 10^9^ colony-forming units per milliliter. The *E. coli* samples were extracted by ExtractPRO Protein Extraction Reagent (Visual Protein, Taiwan) and re-suspended directly in RIPA buffer. Finally, the supernatant was kept at −80 °C.

#### **Gel electrophoresis**

The concentration of protein was determined by the BCA method, and protein samples were analyzed by 12.5% SDS-PAGE. After electrophoresis, the gels were stained with VisPRO 5-min protein stain kit (Visual Protein, Taiwan). After staining, the gels were washed in Milli-Q water.

#### In-gel digestion

The gel lanes corresponding to the bacterial proteins were cut into five slices, and each slice was processed for in-gel digestion according to the method of Shevchenko^25^. Briefly, slices were washed/dehydrated three times in 50 mM ABC (ammonium bicarbonate pH 7.9)/50 mM ABC + 50% ACN (acetonitrile). Subsequently, cysteine bonds were reduced with 10 mM dithiothreitol for 1 h at 56 °C and alkylated with 50 mM iodoacetamide for 45 min at room temperature (RT) in the dark. After two subsequent wash/dehydration cycles, the slices were dried for 10 min in a vacuum centrifuge (ThermoFisher, Breda, The Netherlands) and incubated overnight with 6.25 ng/μL trypsin in 50 mM ABC at 25 °C. Peptides were extracted once in 100 μL of 1% formic acid and subsequently twice in 100 μL of 50% ACN in 5% formic acid. The volume was reduced to 50 μL in a vacuum centrifuge prior to LC-MS/MS analysis.

#### Nano-LC separation and mass spectrometry

Peptides were separated using an Ultimate 3000 nanoLC system (Dionex LC-Packings, Amsterdam, The Netherlands) equipped with a 20 cm × 75 μm i.d. fused silica column custom packed with 3 μm 120 A ReproSil Pur C18 aqua (Dr. Maisch, GMBH, Ammerbuch-Entringen, Germany). After injection, peptides were trapped at 30 μL/min on a 5 mm × 300 μm i.d. Pepmap C18 cartridge (Dionex LCPackings, Amsterdam, The Netherlands) at 2% buffer B (buffer A, 0.05% formic acid in MQ; buffer B, 80% ACN and 0.05% formic acid in MQ) and separated at 300 nL/min in a 10–40% buffer B gradient in 60 min. Eluting peptides were ionized at 1.7 kV in a Nanomate Triversa Chip-based nanospray source using a Triversa LC coupler (Advion, Ithaca, NJ). Intact peptide mass spectra and fragmentation spectra were acquired on a LTQFT hybrid mass spectrometer (Thermo Fisher, Bremen, Germany). Intact masses were measured at a resolution of 50,000 in the ICR cell using a target value of 1 × 106 charges. In parallel, following an FT prescan, the top 5 peptide signals (charge-states 2+ and higher) were submitted to MS/MS in the linear ion trap (3 amu isolation width, 30 ms activation, 35% normalized activation energy, *Q*-value of 0.25 and a threshold of 5000 counts). Dynamic exclusion was applied with a repeat count of 1 and an exclusion time of 30 s.

#### Database search

MS/MS spectra were searched against *E. coli* using Sequest (version 27, rev 12), which is part of the BioWorks 3.3 data analysis package (Thermo Fisher, San Jose, CA). MS/MS spectra were searched with a maximum allowed deviation of 10 ppm for the precursor mass and 1 amu for fragment masses. Methionine oxidation and cysteine carboxamidomethylation were allowed as variable modifications, two missed cleavages were allowed and the minimum number of tryptic termini was 1. After the database search, the DTA and OUT files were imported into Scaffold (versions 1.07 and 2.01) (Proteomesoftware, Portland, OR). Scaffold was used to organize the data and to validate peptide identifications using the Peptide Prophet algorithm and only identifications with a probability >95% were retained. Subsequently, the Protein-Prophet algorithm was applied and protein identifications with a probability of >99% with 2 peptides in at least one of the samples were retained. Proteins that contained similar peptides and could not be differentiated based on MS/MS analysis alone were grouped. For each protein identified, the number of spectral counts (the number of MS/MS associated with an identified protein) is listed in Supplemental Table S5.

### Data analysis

All experiments were performed a minimum of three times independently. Two values were compared with a paired *t*-test, and three or more values of one independent variable was conducted with matched one-way ANOVA with Tukey’s method and more than two independent variables by two-way ANOVA with the Bonferroni post test. All data analysis was performed using SPSS, v. 13.0 (SPSS, Chicago, IL). Statistical significance was set at *P* < 0.05.

## Results

### Functional genomic screening of the EHEC transposon library in *C. elegans*

**Fig. 1:**
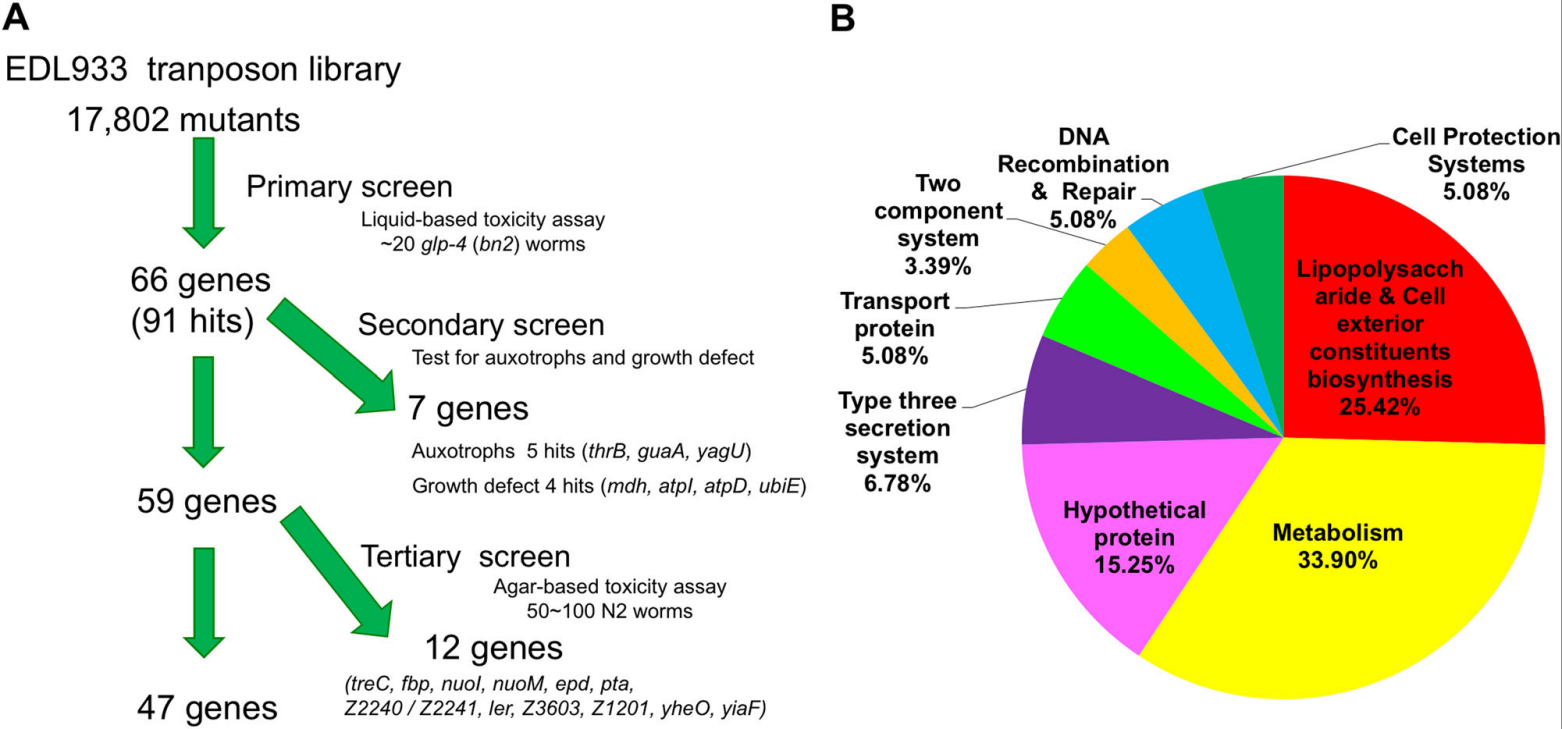
Screening for the attenuated EHEC mutants from the EDL933 transposon mutagenesis library in *C. elegans*. **a** Pipeline of the screen for EDL933 virulence-attenuated mutants in *C. elegans*. **b** The Gene ontology (GO) analysis of these EDL933-attenuated mutants

To identify genes involved in the EHEC pathogenicity in vivo, we conducted a global mutational study by using the EHEC strain EDL933 Tn*5* transposon library. After the primary screen of the 17,802 EDL933 mutants in liquid-based killing assay, 91 hits with a decreased pathogenic phenotype toward *C. elegans* were identified. The transposition sites of Tn*5* in these 91 clones were determined (Fig. 1a and Supplemental Table S6). These 91 hits included mutations in 66 genes. We eliminated 4 hits with growth defect and 5 hits with auxotrophic phenotype after the secondary screen. It has been reported that *Pseudomonas aerginosa* killing *C. elegans* in liquid-based killing assay uses different virulence factors from plate-based assay^26^. We therefore conducted plate-based killing assays to examine the survival rate of *C. elegans* fed with these identified EDL933 mutants. Among the 59 genes, mutations of 47 genes conferred attenuated phenotype after this tertiary screen, which reconfirmed that these genes are required for full EHEC toxicity in both liquid-based and plate-based killing. Interestingly, the functions of these 59 genes were diverse (Fig. 1b). Moreover, several bacterial factors found in our genetic screening have also been reported as important virulence factors for EHEC, including *ler* and *eae*, and *hfq*^27,28^. Given that the genes involved in metabolism represented the major part of our hits, we turned our attention to the genes in this category.

### *sdhA* is required for the pathogenicity of EHEC in *C. elegans*

**Fig. 2:**
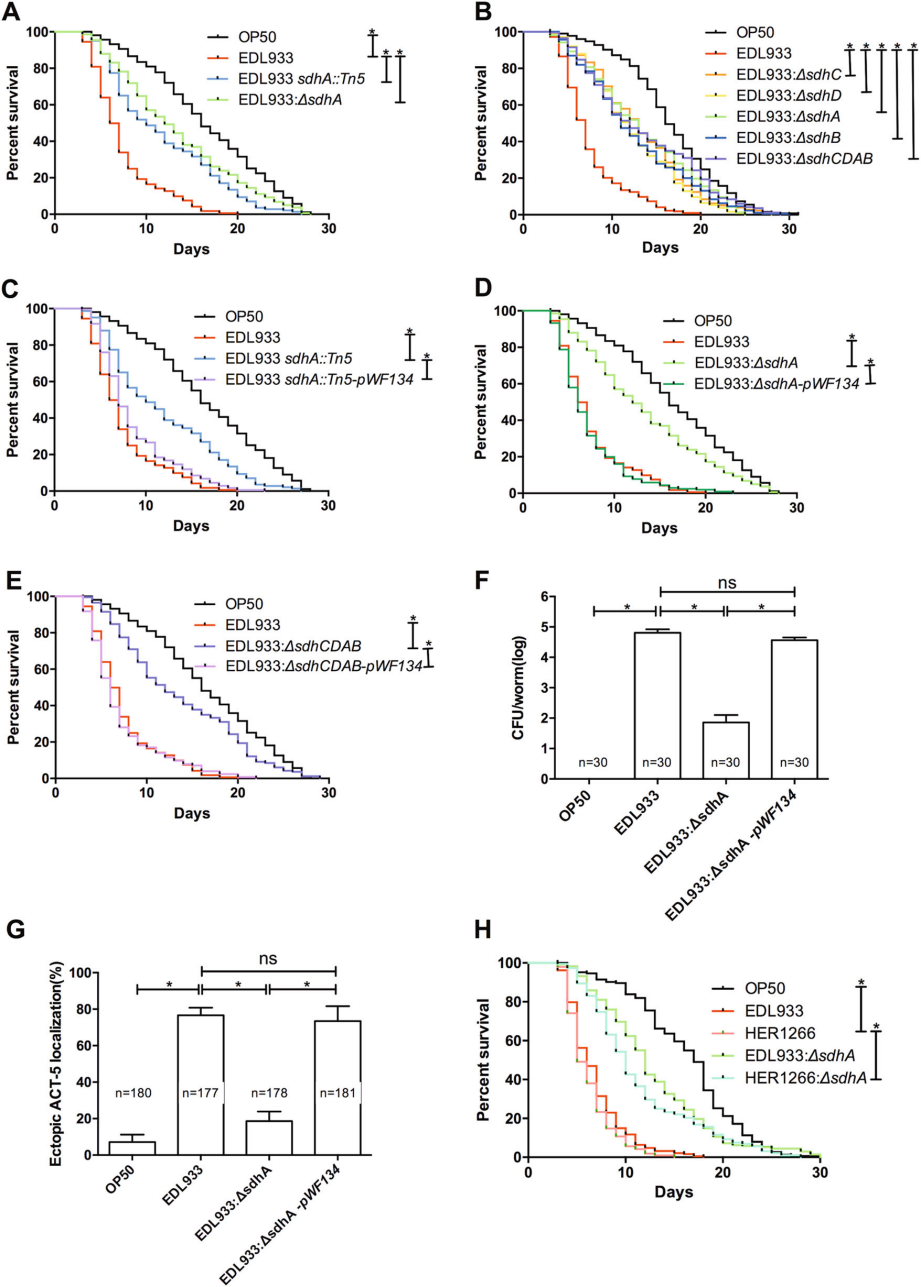
Effect of the *sdhA* mutation on the pathogenicity of EHEC in *C. elegans*. **a** The survival of N2 worms fed with *E. coli* strain OP50, wild-type EHEC strain EDL933, the isogenic transposon-generated mutant strain EDL933 *sdhA::Tn5*, and the isogenic *sdhA* deletion mutant strain EDL933:Δ*sdhA* were examined. The EDL933 *sdhA::Tn5* strain (median N2 lifespan = 10.5 ± 1.5 days, *P* < 0.0001) and the EDL933:Δ*sdhA* strain (median N2 lifespan = 12.0 ± 2.0 days, *P* < 0.0001) were both significantly less toxic than the wild-type EDL933 strain (median N2 lifespan = 6.5 ± 0.5 days). **b** The survival of N2 worms fed with OP50, wild-type EDL933, and EDL933 with deletion of *sdhC*, *sdhA*, *sdhD*, *sdhB*, or *sdhCDAB* operon were examined. Deletion of *sdhC* (EDL933:Δ*sdhC*) (median N2 lifespan = 13.0 ± 2.0 days, *P* < 0.0001), *sdhD* (EDL933:Δ*sdhD*) (median N2 lifespan = 11.5 ± 1.5 days, *P* < 0.0001), *sdhB* (EDL933:Δ*sdhB*) (median N2 lifespan = 12.0 ± 1.0 days, *P* < 0.0001) genes, and the entire *sdhCDAB* operon (EDL933:Δ*sdhCDAB*) (median N2 lifespan = 12.5 ± 2.5 days, *P* < 0.0001) were all significantly less toxic than the wild-type EHEC strain EDL933 (EDL933) (median N2 lifespan = 6.75 ± 0.25 days). **c–e** The survival of N2 worms fed with *sdhA* transposon-generated mutant EDL933 *sdhA::Tn5*, *sdhA* deletion mutant (EDL933:Δ*sdhA*), *sdhCDAB* deletion mutant (EDL933:Δ*sdhCDAB*), and isogeneic strains rescued by the transformation of the *sdhCDAB* operon expression plasmid *pWF134* (EDL933 *sdhA::Tn5*-*pWF134*, EDL933:Δ*sdhA-pWF134*, EDL933:Δ*sdhCDAB-pWF134*, respectively) were examined. **c** The decreased pathogenic phenotype in the EDL933 *sdhA::Tn5* (median N2 lifespan = 10.5 ± 1.5 days) was restored by the *sdhA* gene complementation EDL933 *sdhA::Tn5*-*pWF134* (median N2 lifespan = 7.0 ± 0.1 days, *P* < 0.0001). **d** EDL933:Δ*sdhA* (median N2 lifespan = 12.00 ± 2.00 days) and EDL933:Δ*sdhA*-*pWF134* (median N2 lifespan = 6.0 ± 1.0 days, *P* < 0.0001). **e** EDL933:Δ*sdhCDAB* (median N2 lifespan = 12.5 ± 2.5 days) and EDL933:Δ*sdhCDAB*-*pWF134* (median N2 lifespan = 5.5 ± 0.5 days, *P* < 0.0001). The median lifespan of N2 worms fed with wild-type EDL933 was 6.5 ± 0.5 days. **f** The number of bacteria colonized in *C. elegans* was determined by the colony-forming units (CFUs) assay from three independent experiments. The average CFU per infected worm fed with the *sdhA* deletion mutant was significantly decreased (7.10 × 10^1^, *P* < 0.0001) compared to that of the wild-type EDL933 infected animals (6.43 × 10^4^). The average CFU per infected worm fed with the *sdhA* gene complementation strain (3.65 × 10^4^, *P* = 0.1) was similar to that of the wild-type EDL933 infected animals. The asterisk denotes statistically significant (*P* < 0.0001) examined by the *t*-test, and error bars indicate the SEM (standard error of mean) of three independent experiments. The total numbers of infected animals examined in each group are indicated by *n*. **g** Quantification of the ectopic mCherry::ACT-5 signal in GK454 nematodes exposed to OP50, EDL933, *sdhA* deletion mutant (EDL933:Δ*sdhA*), or *sdhA* gene complementation strain (EDL933:Δ*sdhA*-*pWF134*) at 20 °C for 4 days. The asterisk denotes statistically significant (*P* < 0.0001) examined by the *t*-test, and error bars indicate the SEM of three independent experiments. The total numbers of infected animals examined in each group are indicated by *n*. **h** Survival of N2 worms fed with the *E. coli* O157:H7 strain HER1266 and the isogenic strain with *sdhA* deletion. Deletion of *sdhA* in HER1266 (HER1266*:*Δ*sdhA*) confers the attenuated toxic phenotype (median lifespan of N2 animals = 10.0 ± 0.1 days, *P* < 0.0001). The median lifespan of N2 worms fed with wild-type HER1266 was 5.50 ± 0.5 days

We noted that several genes in central metabolism, especially the succinate dehydrogenase (*sdh*) genes, are required for the pathogenesis of EHEC in *C. elegans*. The transposon mutant *sdhA::Tn5*, strain YQ413 (ED97-A-1 in Supplemental Table S6) conferred a significant decrease in the virulence toward *C. elegans* (Fig. 2a). In order to reconfirm that mutation in *sdhA* attenuates the toxicity of EHEC, an isogenic deletion mutant (EDL933:Δ*sdhA*) was generated. The *sdhA* deletion significantly attenuated the pathogenicity of EHEC compared to the parental wild-type EDL933 (Fig. 2a). Of note, the *sdhA* mutants, including the transposon and deletion mutants, all showed no growth defect (Supplemental Figure S2), which suggested that *sdhA* may play unidentified roles in EHEC pathogenicity. Hence, we turned our focus to the mechanism of *sdhA* in the regulation of EHEC infection.

The Sdh complex consists of four subunits and is encoded by the *sdhCDAB* operon^29^. We found that disruptions in any genes of the complex, including *sdhC*, *sdhD*, *sdhA*, and *sdhB*, and the entire *sdhCDAB* operon all conferred attenuated toxicity of EHEC toward *C. elegans* (Fig. 2b). These data suggest that Sdh activity is required for the pathogenesis of EHEC. In order to reconfirm the role of Sdh in the virulence of EHEC, the *sdhCDAB* operon expression plasmid *pWF134* was generated for complementation of these mutants. Complementation of the *sdh* mutations by the *pWF134* reversed the attenuated toxicity of the transposon-generated *sdhA* mutant EDL933 s*dhA::Tn5*, (Fig. 2c), the *sdhA* deletion mutant EDL933:Δ*sdhA* (Fig. 2d), and the *sdhCDAB* operon deletion mutant EDL933:Δ*sdhCDAB* (Fig. 2e). Taken together, our genetic analysis demonstrated that the *sdh* genes, including *sdhA*, are required for the virulence of EHEC strain EDL933 in *C. elegans*.

Our previous study demonstrated that EHEC can colonize and induce abnormal microvillus morphology, a biomarker of the A/E lesion, in the intestine of *C. elegans*^19^. Here we demonstrated that mutation of the EHEC *sdhA* gene significantly reduced its colonization in *C. elegans* (Fig. 2f). Moreover, complementation by *pWF134* restored this phenotype of the EDL933:Δ*sdhA* mutant. The abnormal microvillus morphology induced by EHEC can be detected by the mislocalization of the intestine microvillar Actin-5 that is normally expressed on the apical site and in the microvilli of intestinal cells^19^. Compared to the wild-type EDL933, the EHEC-induced ectopic microvillar actin localization was significantly diminished in the EDL933:Δ*sdhA* infected *C. elegans* animals (Fig. 2g). Moreover, complementation of the *sdhA* mutation by *pWF134* significantly rescued the phenotype. Together, our results demonstrated that *sdhA* mutation decreased the virulence of EHEC toward *C. elegans*.

In order to test whether *sdhA* is also a virulence determinant for the other EHEC strain, an isogenic mutant with *sdhA* deletion in the EHEC strain HER1266 (HER1266:Δ*sdhA*) was generated. Disruption of the *sdhA* gene also attenuated the toxicity of HER1266 in *C. elegans* (Fig. 2h). However, *C. elegans* fed on OP50:Δ*sdhA* exhibited a similar survival curve to that of the worms fed on wild-type OP50, the normal food source of *C. elegans* (Supplemental Figure S4B). Together, our data indicated that *sdhA* is a specific virulence determinant and required for EHEC infection in *C. elegans*.

### Deletions of the TCA cycle genes confer the attenuated virulence of EHEC

**Fig. 3:**
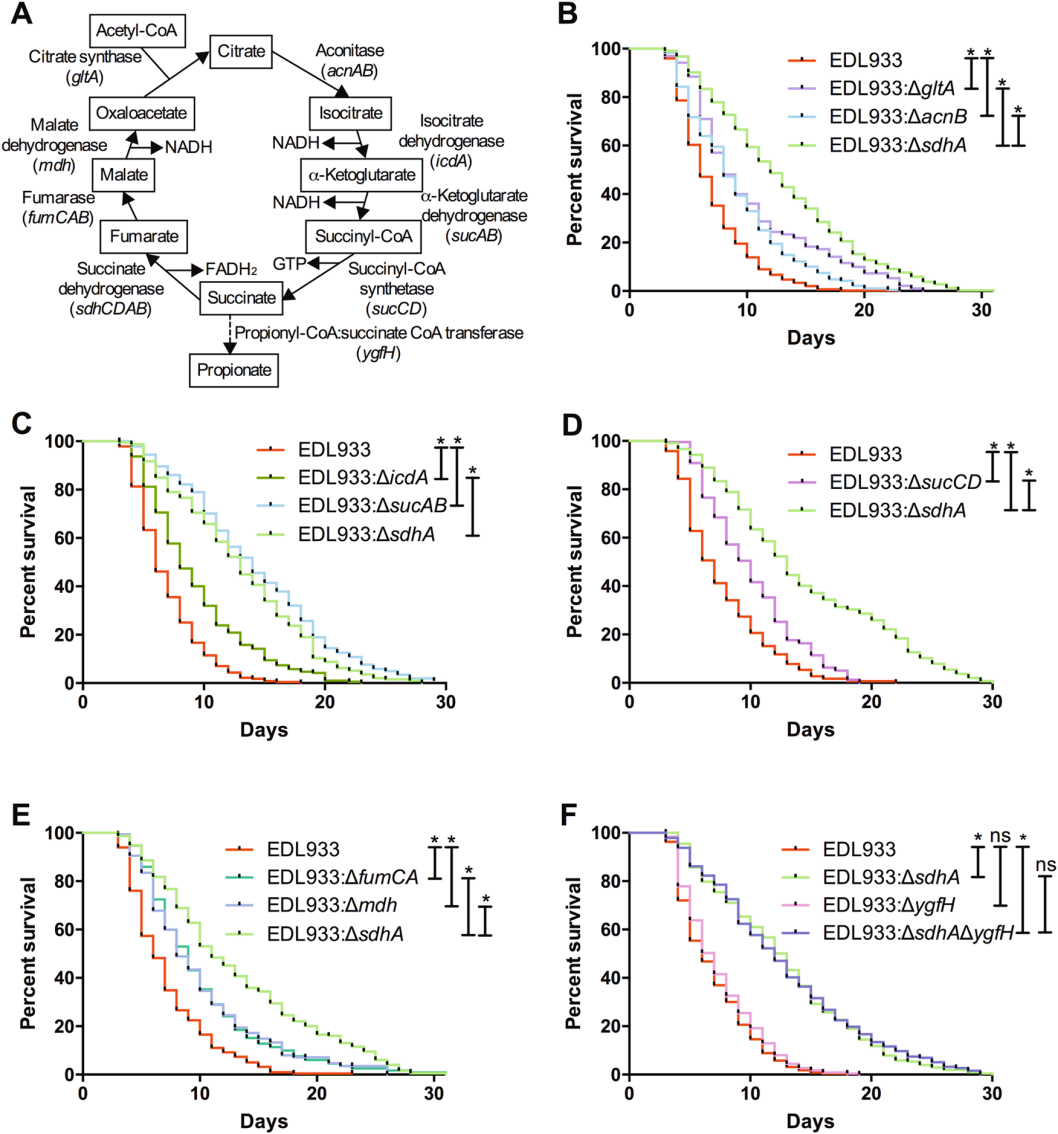
Effect of the incomplete TCA cycle and succinate metabolism on the virulence of EHEC. **a** A diagram of the enzymes (genes) and metabolites of the TCA cycle and the alternative metabolic path of succinate through YgfH. **b** The survival of N2 worms fed with the wild-type EHEC strain EDL933 (EDL933) and the isogenic EDL933 strains with deletion of *gltA* (EDL933*:*Δ*gltA*), *acnB* (EDL933*:*Δ*acnB)*, and *sdhA* (EDL933:Δ*sdhA*) were examined. Deletions of *gltA* (median N2 lifespan = 8.5 ± 0.7 days, *P* < 0.0001), *acnB* (median N2 lifespan = 8.0 ± 0.1 days, *P* < 0.0001), and *sdhA* (median N2 lifespan = 12.0 ± 2.2 days, *P* < 0.0001) all conferred the attenuated toxic phenotype compared to the wild-type EDL933 (the median N2 lifespan = 6.0 ± 0.1 days). Moreover, EDL933:Δ*sdhA* mutant was significantly less toxic compared to the EDL933*:*Δ*gltA* (*P* < 0.0001) and EDL933*:*Δ*acnB* (*P* < 0.0001). **c** The survival of N2 worms fed with the wild-type EDL933 and the isogenic deletion strains of *icdA* (EDL933*:*Δ*icdA*), *sucAB* (EDL933*:*Δ*sucAB)*, and *sdhA* (EDL933:Δ*sdhA*) were examined. Deletions of *icdA* (median N2 lifespan = 9.0 ± 1.4 days, *P* < 0.005), *sucAB* (median N2 lifespan = 13.5 ± 2.1 days, *P < *0.0001), and *sdhA* (median N2 lifespan = 13.5 ± 2.1 days, *P < *0.0001) all conferred the attenuated toxic phenotype compared to the wild-type EDL933 (the median N2 lifespan = 6 ± 0.2 days). The EDL933:Δ*sdhA* mutant was significantly less toxic compared to both the EDL933*:*Δ*icdA* (*P* < 0.0001) and the EDL933*:*Δ*sucAB* mutant (*P < *0.05). **d** The survival of N2 worms fed with the wild-type EDL933 and the isogenic deletion strains of *sucCD* (EDL933*:*Δ*sucCD*) and *sdhA* (EDL933:Δ*sdhA*) were examined. Deletion of *sucCD* (median N2 lifespan = 9.0 ± 1.0 days, *P* < 0.0001) and *sdhA* (median N2 lifespan = 11.3 ± 1.5 days, *P* < 0.0001) conferred the attenuated toxic phenotype compared to the wild-type EDL933 (the median N2 lifespan = 6.3 ± 0.5 days). Moreover, EDL933:Δ*sdhA* mutant was significantly less toxic compared to the EDL933*:*Δ*sucCD* (*P* < 0.0001). **e** The survival of N2 worms fed with the wild-type EDL933 and the isogenic deletion strains of *fumCA* (EDL933*:*Δ*fumCA*), *mdh* (EDL933*:*Δ*mdh)*, and *sdhA* (EDL933:Δ*sdhA*) were examined. Deletions of *fumCA* (median N2 lifespan = 9.0 ± 0.1 days, *P* < 0.0001), *mdh* (median N2 lifespan = 8.5 ± 0.5 days, *P* < 0.0001), and *sdhA* (median N2 lifespan = 10.5 ± 0.5 days, *P* < 0.0001) all conferred the attenuated toxic phenotype compared to the wild-type EDL933 (median N2 lifespan = 6.1 ± 0.1 days). Moreover, EDL933:Δ*sdhA* mutant was significantly less toxic compared to the EDL933*:*Δ*fumCA* (*P < *0.0001) and EDL933*:*Δ*mdh* (*P* < 0.0001). **f** The survival of N2 worms fed with the wild-type EDL933 and the isogenic deletion strains of *ygfH* (EDL933*:*Δ*ygfH)*, *sdhA* (EDL933*:*Δ*sdhA*), and the *ygfH* and *sdhA* double mutant (EDL933:Δ*ygfH*Δ*sdhA*) were examined. Deletion of *ygfH* (median N2 lifespan = 6.3 ± 0.4 days, *P* = 0.102) did not attenuate its toxicity. Although deletion of the *ygfH* and *sdhA* double mutant (EDL933:Δ*ygfH*Δ*sdhA*, median N2 lifespan = 11.5 ± 0.7 days, *P* < 0.0001) conferred the attenuated toxic phenotype compared to the wild-type EDL933 (the median N2 lifespan = 5.0 ± 0.5 days), the virulence of EDL933:Δ*ygfH*Δ*sdhA* strain was similar to the EDL933*:*Δ *sdhA* mutant (median N2 lifespan = 12.5 ± 0.7 days, *P* = 0.691)

Sdh converts succinate to fumarate in the TCA cycle (Fig. 3a). In addition to the *sdhCDAB* operon, the *gltA, acnAB, icdA, sucAB, sucCD, fumCAB*, and *mdh* genes encode for the other enzymes in the cycle. To examine whether the TCA cycle is required for the virulence regulation of EHEC, we interrupted the TCA cycle by generating isogeneic deletion mutants of these genes. Our data demonstrated that deletion of these genes significantly attenuated the toxicity of EHEC toward *C. elegans* (Fig. 3b–e). Interestingly, the effects of disruption of these TCA cycle genes (*gltA, acnB, icdA, sucAB, sucCD, fumCA*, and *mdh* respectively) in EHEC virulence attenuation were all significantly less (all *P < *0.05) than the disruption of the *sdhA* gene. These results suggested that disruption of the TCA cycle accounts only partly for the attenuated virulent phenotype of the *sdhA* mutant. Nevertheless, our data suggested that the TCA cycle plays an important role in the pathogenesis of EHEC.

Except for Sdh, which oxidized succinate to form fumarate, propionyl-CoA:succinate CoA transferase, encoded by the *ygfH* gene, can also decarboxylate succinate to form propionate (Fig. 3a). However, we found that disruption of the *ygfH* gene had no effect on the virulence of EHEC toward *C. elegans* (Fig. 3f). Moreover, the *ygfH* and *sdhA* double mutant conferred the same attenuated toxic phenotype as the *sdhA* single mutant. Our genetic data indicated that *ygfH* is not required for the EHEC virulence in *C. elegans*. Nevertheless, our results reconfirmed that *sdhA* is required for full toxicity of EHEC in *C. elegans*.

### EHEC attenuates its toxicity upon anaerobic respiration

*E. coli* cells have three metabolic modes of energy metabolism: aerobic respiration, anaerobic respiration, and fermentation. Sdh also serves as the complex II in the aerobic respiratory chain. Given that interruption of the TCA cycle by deletion of *sdhA* decreases the toxicity of EHEC, we hypothesized that EHEC is more toxic upon oxybiosis. To this end, we constructed isogenic deletion mutants of the genes encoded for the enzymes and regulators in either aerobic respiration or anaerobic metabolism of EHEC, and tested their toxicity toward *C. elegans*.

**Fig. 4:**
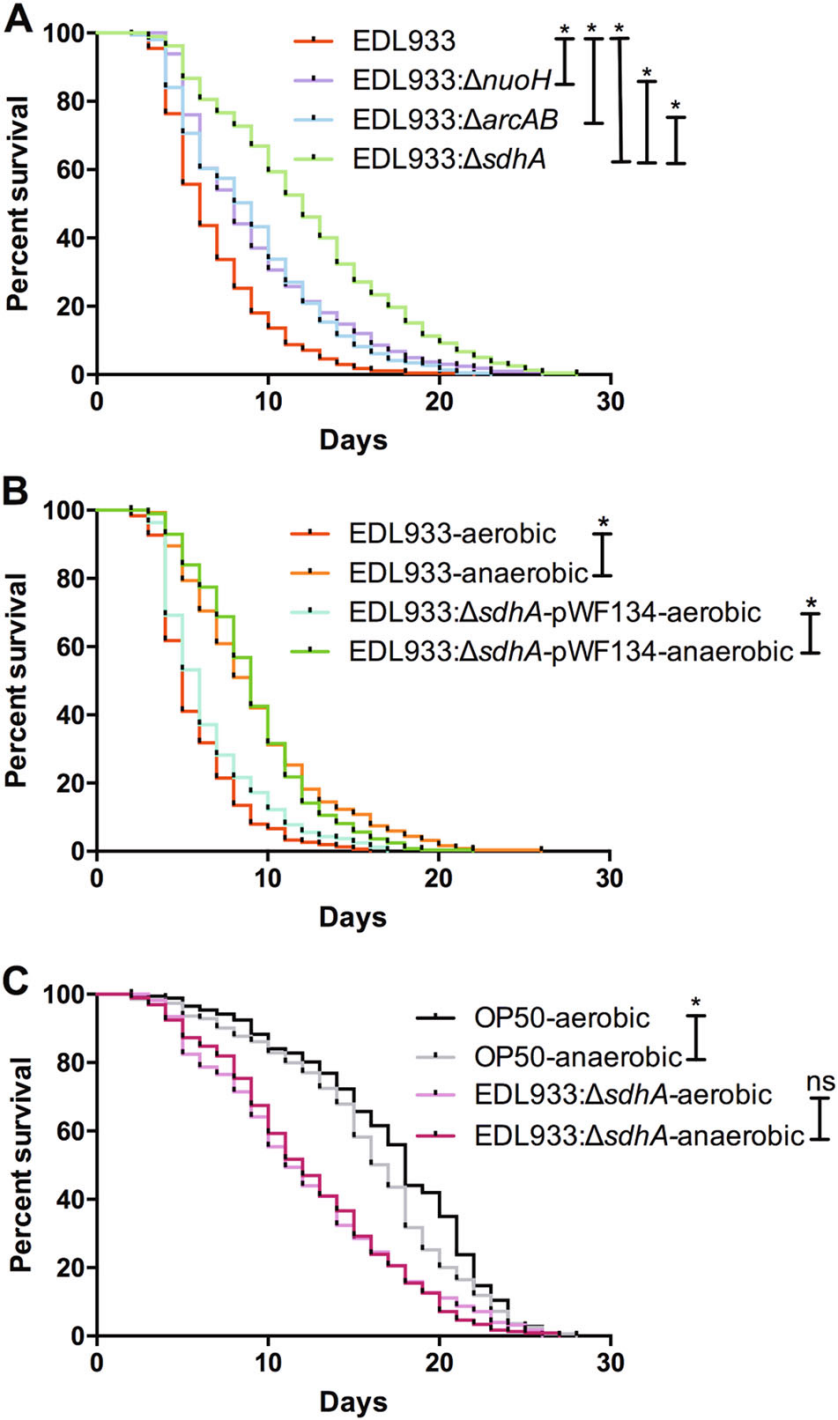
Effect of aerobic respiration on the pathogenicity of EHEC. **a** The survival of N2 worms fed with the EHEC wild-type strain EDL933 (EDL933) and the isogenic deletion strains of *nuoH* (EDL933*:*Δ*nuoH*), *arcAB* (EDL933*:*Δ*arcAB*), and *sdhA* (EDL933:Δ*sdhA*) were examined. Deletions of *nuoH* (median N2 lifespan = 8 ± 0.1 days, *P* < 0.0001), *arcAB* (median N2 lifespan = 10 ± 0.1 days, *P < *0.0001), and *sdhA* (median N2 lifespan = 12.5 ± 0.7 days, *P* < 0.0001) all conferred the attenuated toxic phenotype compared to the wild-type EDL933 (median N2 lifespan = 6.3 ± 0.5 days). The EDL933:Δ*sdhA* mutant was significantly less toxic compared to both the EDL933*:*Δ*nuoH* (*P* < 0.0001) and EDL933*:*Δ*arcAB* (*P < *0.0001). **b** The survival of N2 worms fed with wild-type EDL933 (EDL933) and EDL933:Δ*sdhA*-*pWF134* strain which were cultured aerobically or anaerobically were examined. The EDL933 strain when cultured anaerobically (EDL933-anaerobic, median N2 lifespan = 9.0 ± 1.4 days, *P* < 0.0001) conferred the attenuated toxic phenotype compared to the same strain cultured aerobically (EDL933-aerobic, median N2 lifespan = 5.5 ± 0.7 days), and the EDL933:Δ*sdhA*-*pWF134* strain when cultured anaerobically (EDL933:Δ*sdhA*-*pWF134*-anaerobic, median N2 lifespan = 9.0 ± 0.1 days, *P* < 0.0001) also conferred the attenuated toxic phenotype compared to the same strain cultured aerobically (EDL933:Δ*sdhA*-*pWF134*-aerobic, median N2 lifespan = 5.5 ± 0.7 days). **c** The survival of N2 worms fed with the wild-type OP50 strain (OP50) and the EDL933:Δ*sdhA* strain, cultured aerobically or anaerobically, were examined. Worms fed on anaerobic cultured OP50 (OP50-anaerobic, median N2 lifespan = 16.5 ± 0.7 days, *P* < 0.05) exhibited significant shorter lifespan compared to that on the aerobic culture control group (OP50-aerobic, median N2 lifespan = 18 ± 0.1 days). The survival of N2 worms fed on anaerobic cultured EDL933:Δ*sdhA* (EDL933:Δ*sdhA-*anaerobic, median N2 lifespan = 13.0 ± 2.8 days, *P* = 0.980) was similar to that on the same strain cultured aerobically (EDL933:Δ*sdhA-*aerobic, median N2 lifespan = 12.0 ± 1.4 days)

Besides Sdh, the NADH dehydrogenase (complex I), encoded by the *nuo* operon, can transfer electrons to ubiquinone for aerobic respiration. Our results showed that the survival curve of *C. elegans* fed with EDL933:Δ*nuoH* was significantly longer than that of animals fed with the wild-type EDL933 (Fig. 4a). Moreover, the ArcAB two-component regulatory system is the major regulator of aerobic respiration^6,30^. Our data indicated that the *arcAB* double mutant (EDL933:Δ*arcAB*) strain was significantly less toxic compared to the wild type (Fig. 4a). However, both the EDL933:Δ*nuoH* and EDL933:Δ*arcAB* mutants were significantly more toxic than the EDL933:Δ*sdhA* mutant. The results suggested that disruption of aerobic respiration partly accounts for the attenuated virulent phenotype of the *sdhA* mutant. Moreover, we also demonstrated that anaerobic metabolism is dispensable for the full virulence of EHEC in *C. elegans* (Supplemental Figure S3). Taken together, these results supported the notion that EHEC is more toxic when utilizing aerobic respiration.

To test the hypothesis further, we cultured the EDL933 bacterial cells either aerobically or anaerobically and tested their toxicity toward *C. elegans*. We observed that the virulence of the wild-type EDL933 and the EDL933:Δ*sdhA*-*pWF134* strains when cultured anaerobically were both significantly attenuated compared to the same strains cultured aerobically (Fig. 4b). These data support the notion that EHEC is more toxic in aerobic metabolism. Moreover, *C. elegans* fed on EDL933:Δ*sdhA* cells cultured anaerobically exhibited similar survival curves to worms fed on the bacterial cells cultured aerobically (Fig. 4c). Together, our results demonstrated that aerobic respiration plays a role in the regulation of pathogenicity of EHEC in *C. elegans*, and accounts partly for the decreased virulence of the *sdhA* mutant.

### The depletion of fumarate resulted from *sdhA* mutation affecting the EHEC toxicity

**Fig. 5:**
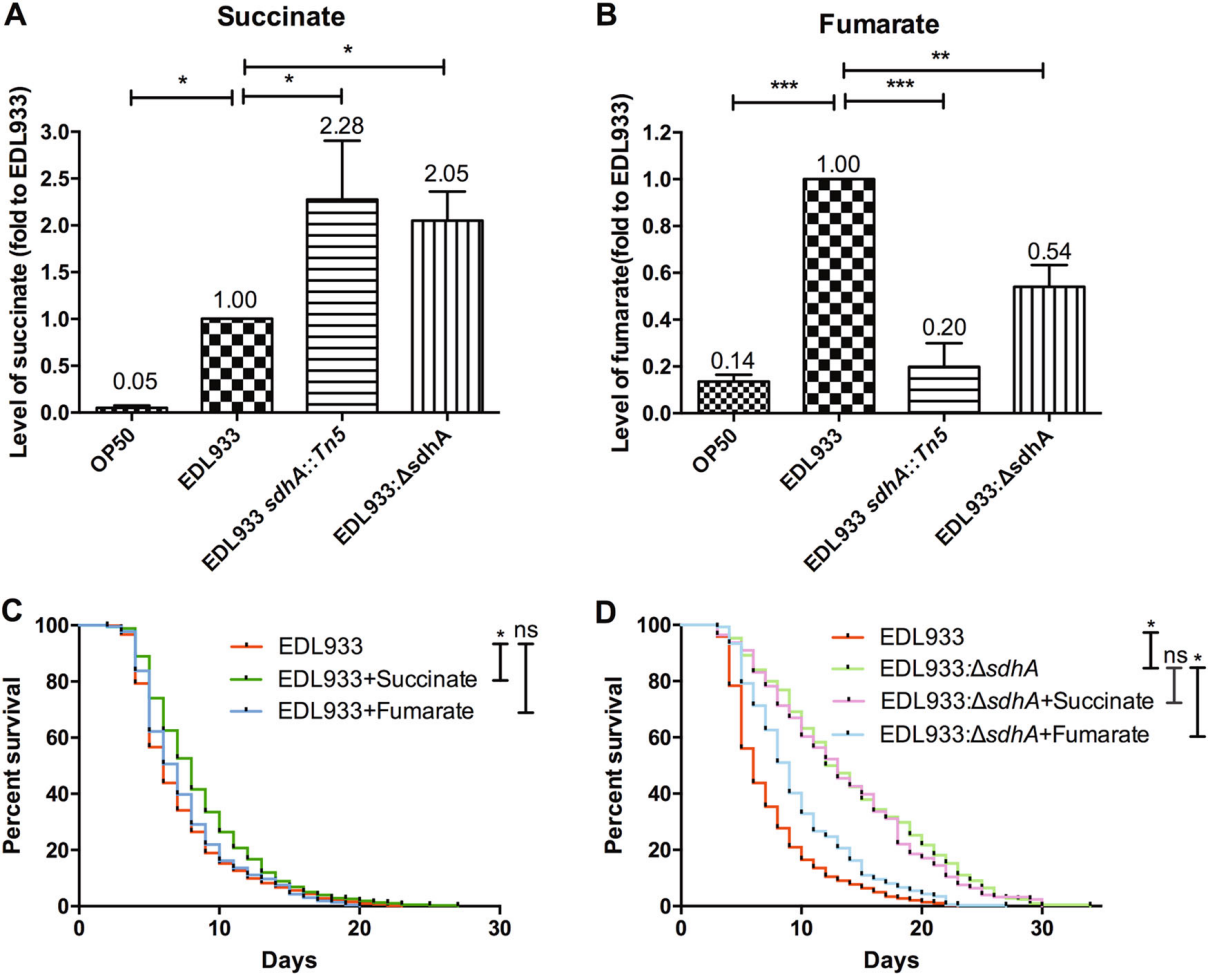
Effect of metabolite alteration on the pathogenesis of EDL933:Δ*sdhA* mutant. **a** The relative level of succinate in OP50 strain, wild-type EDL933 strain, *sdhA* transposon-generated mutant strain EDL933 *sdhA::Tn5*, and *sdhA* deletion mutant strain EDL933:Δ*sdhA* were determined. The asterisk denotes statistical significance (*P* < 0.0001) as examined by the *t*-test, and error bars indicate the SEM (Standard Error of the Mean) of three independent experiments. **b** The relative level of fumarate in OP50, EDL933, EDL933 *sdhA::Tn5*, and EDL933:Δ*sdhA* were determined. **c** The survival curves of N2 worms fed with the wild-type EDL933 strain only (EDL933), EDL933 cultured in media supplemented with 2.5 mM succinate (EDL933 + Succinate), or 2.5 mM fumarate (EDL933 + Fumarate) were examined. Treatment of succinate in EDL933 strain conferred the attenuated toxic phenotype (EDL933 + Succinate, median N2 lifespan = 7.8 ± 0.6 days, *P* < 0.0001) compared to the untreated wild-type EDL933 strain control (median N2 lifespan = 5.9 ± 0.3 days). Treatment of fumarate in EDL933 strain expressed similar toxicity (median N2 lifespan = 6.7 ± 0.3 days, *P* = 0.18) as the untreated wild-type EDL933 strain control (EDL933 + Fumarate, N2 median lifespan = 5.9 ± 0.3 days). **d** The survival curves of worms fed with the wild-type EDL933 strain (EDL933), and the isogenic deletion strains of *sdhA* (EDL933:Δ*sdhA*) with 2.5 mM succinate (EDL933:Δ*sdhA* + Succinate) or 2.5 mM fumarate (EDL933:Δ*sdhA + *Fumarate) were tested. The survival curve of worms fed on succinate-treated EDL933:Δ*sdhA* (EDL933:Δ*sdhA* + Succinate, median N2 lifespan = 12.5 ± 2.1 days, *P* = 0.927) was similar to that on the control EDL933:Δ*sdhA* (EDL933:Δ*sdhA*, median N2 lifespan = 13.3 ± 1.5 days). However, treatment of fumarate in EDL933:Δ*sdhA* mutant (EDL933:Δ*sdhA + *Fumarate, median N2 lifespan = 8.8 ± 0.8 days, *P* < 0.0001) significantly reversed the toxicity attenuation of the EDL933:Δ*sdhA* mutant (EDL933:Δ*sdhA*, N2 median lifespan = 13.3 ± 1.5 days)

A major metabolic function of Sdh is to convert succinate into fumarate. We surmised that the alteration in the concentrations of related metabolites could potentially influence the toxicity of EHEC. Thus, we first assessed the level of succinate and fumarate in the EDL933:Δ*sdhA* mutants. We noted that the levels of succinate in the transposon-generated *sdhA* mutant [YQ413 (EDL933 s*dhA::Tn5*)] and the isogenic *sdhA* deletion mutant (EDL933:Δ*sdhA*) of EDL933 strain were both significantly increased compared to the parental EDL933 strain (Fig. 5a). In contrast, the levels of fumarate in the EDL933 s*dhA::Tn5*, and the EDL933:Δ*sdhA* strains were both significantly decreased compared to the wild-type (Fig. 5b). These data reconfirmed that disruption of the enzyme activity of Sdh in the *sdhA* mutants alters the concentrations of the related metabolites in EHEC.

We then tested whether accumulation of succinate or depletion of fumarate caused by loss-of-function of *sdhA* could affect the EHEC pathogenicity. To this end, we cultured wild-type OP50, OP50:Δ*sdhA*, wild-type EDL933 and the EDL933:Δ*sdhA* mutant with 2.5 mM succinate or 2.5 mM fumarate, and tested their toxicity against *C. elegans*. We noted that while treatment of succinate slightly decreased the toxicity of EHEC against *C. elegans*, treatment of fumarate in the wild-type EDL933 had no effect on its virulence (Fig. 5c). However, the toxic attenuation of the EDL933:Δ*sdhA* mutant was significantly reversed by culture of bacterial cells with fumarate, but not with succinate, compared to the untreated EDL933:Δ*sdhA* control (Fig. 5d). The data demonstrated fumarate depletion accounts significantly for the attenuated virulent phenotype of the *sdhA* mutant. We also demonstrated that the effect of fumarate was on EDL933:Δ*sdhA* mutant *per se*, rather than toward *C. elegans* (Supplemental Figure S4). Taken together, our data suggest that fumarate may function as a virulent determinant to regulate the pathogenicity of EHEC.

### TnaA tryptophanase is a downstream virulence effector of *sdhA*

**Fig. 6:**
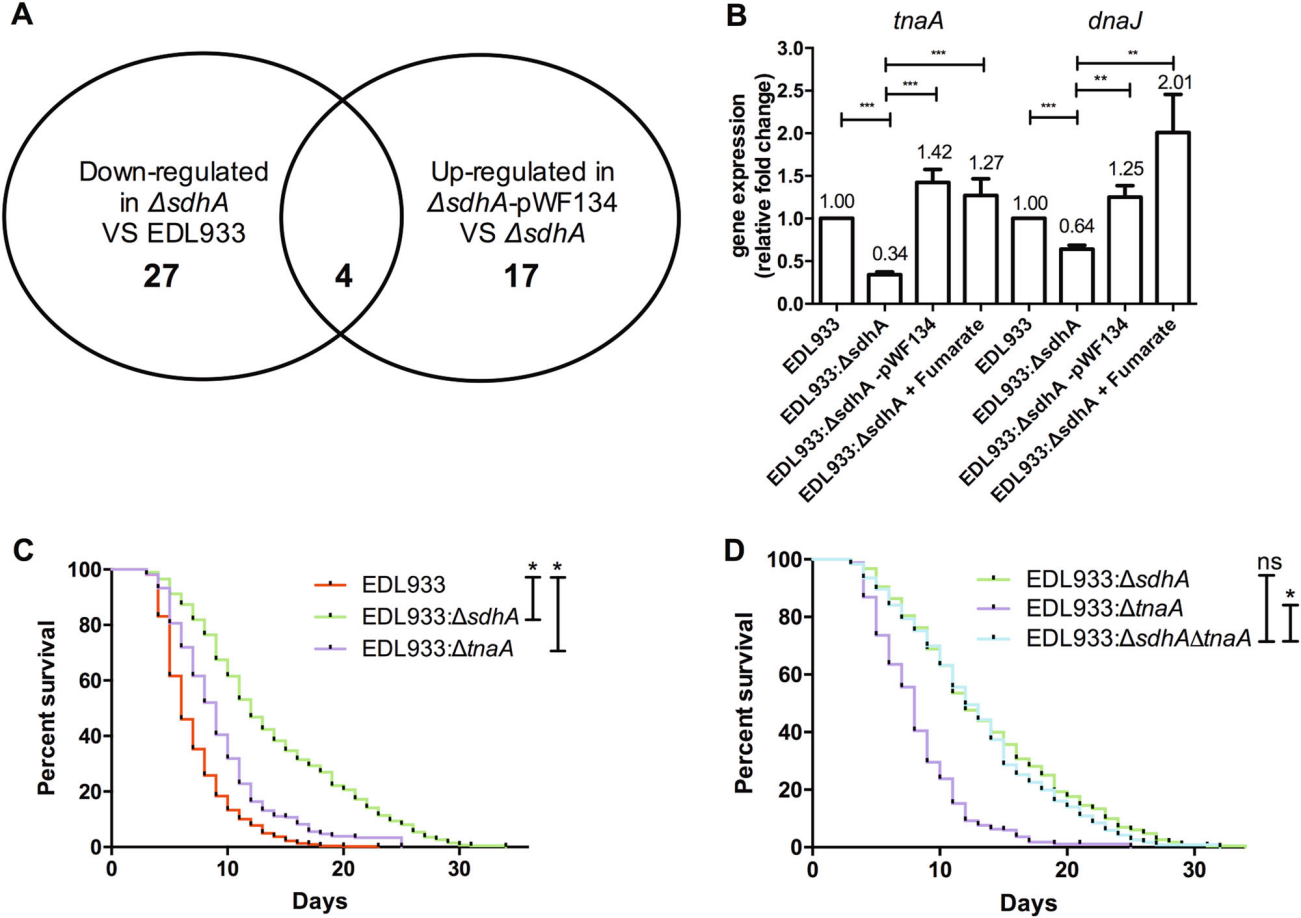
TnaA is a downstream virulence effector of *sdhA* identified by proteomic analysis. **a** The diagram shows that four differentially expressed proteins, which downregulated in the *sdhA* mutant (EDL933:Δ*sdhA*) and upregulated in the *sdhA* complement strain (EDL933:Δ*sdhA*-*pWF134*), were identified by our proteomic analysis. **b** The mRNA levels of *tnaA* and *dnaJ* in EHEC wild-type strain (EDL933), EDL933:Δ*sdhA* mutant strain (EDL933:Δ*sdhA*) the *sdhA* gene complementation strain (EDL933:Δ*sdhA*-*pWF134*) and 2.5 mM fumarate-treated *sdhA* (EDL933:Δ*sdhA* + Fumarate) were examined. Relative transcriptional expression was normalized to the expression of *rpoA*. The asterisk denotes statistically significant (*P* < 0.0001) examined by the *t*-test, and error bars indicate the SEM of three independent experiments. **c** The survival of N2 worms fed with the wild-type strain (EDL933) and the isogenic deletion strains of *tnaA* (EDL933*:*Δ*tnaA*) and *sdhA* (EDL933:Δ*sdhA*) were examined. Deletions of *tnaA* (median N2 lifespan = 8.0 ± 0.1 days, *P* < 0.0001) and *sdhA* (median N2 lifespan = 12.4 ± 1.8 days, *P* < 0.0001) all conferred the attenuated toxic phenotype compared to the wild-type EDL933 (median N2 lifespan = 6.4 ± 0.9 days). **d** The survival of N2 worms fed with the wild-type strain (EDL933) and the isogenic deletion strains of *tnaA* (EDL933*:*Δ*tnaA*), *sdhA* (EDL933*:*Δ*sdhA)*, and the *sdhA* and *tnaA* double mutant (EDL933:Δ*sdhA*Δ*tnaA*) were examined. The virulence of *sdhA* and *tnaA* double mutant (EDL933:Δ*sdhA*Δ*tnaA*, median N2 lifespan = 13.5 ± 0.7 days) was similar to the EDL933*:*Δ*sdhA* (median N2 lifespan = 13.5 ± 2.1 days, *P* = 0.474). The asterisk denotes statistically significant (*P* < 0.0001) examined by the log-rank test, and “ns” represents no significant difference statistically

In order to identify the downstream virulence effectors regulated by the activity of Sdh, we deployed a proteomic analysis to characterize the proteomic changes of EDL933:Δ*sdhA* mutant (Supplemental Table S5). Four differentially expressed proteins, which were downregulated in the *sdhA* mutant as well upregulated in the *sdhA* complement strain, were identified (Fig. 6a and Table 1). These four proteins are the succinate dehydrogenase flavoprotein subunit SdhA, the succinate dehydrogenase iron-sulfur subunit SdhB, the tryptophanase TnaA, and the molecular protein chaperone DnaJ. The identification of the SdhA and SdhB proteins from our analyses validated the reliability of our proteomic data. In addition, we identified two potential Sdh-dependent and downstream virulence modulators, TnaA and DnaJ. Consistent with their expression at the protein level, the transcription levels of *tnaA* and *dnaJ* genes were significantly reduced in the EDL933:Δ*sdhA* mutant compared to the wild-type EDL933 (Fig. 6b). Moreover, the complementation of *sdhA* mutation by *pWF134* transformation as well as fumarate supplement can both restore their mRNA expression in the *sdhA* mutant, which suggested that the expression of *tnaA* and *dnaJ* is regulated in a *sdhA-* and fumarate*-*dependent manner at the transcriptional level.

**Table 1:** Proteins with differential expression in the wild-type EHEC strain (EDL933), the isogenic *sdhA* deletion mutant (EDL933*:*Δ*sdhA*), and the *sdhA* gene complementation strain (EDL933:Δ*sdhA*-*pWF134*)

Protein Name	Gene name	Δ*sdhA*/EDL933			Δ*sdhA*-*pWF134*/Δ*sdhA*		
		*P* value	Fold change	Trend	*P* value	Fold change	Trend
Succinate dehydrogenase flavoprotein subunit	*sdhA*	0.001	−9.45	**↓**	0.014	70.92	**↑**
Succinate dehydrogenase iron-sulfur subunit	*sdhB*	0.002	−10.31	**↓**	<0.0001	65.79	**↑**
Tryptophanase	*tnaA*	0.010	−1.77	**↓**	0.008	2.27	**↑**
Molecular chaperone	*dnaJ*	0.010	−100	**↓**	<0.0001	100	**↑**

Given that *dnaJ* is an essential gene required for *E. coli* growth^31^, we turned our attention to the function *tnaA* gene in the virulence of EHEC. Our results showed that disruption of the *tnaA* significantly attenuated the toxicity of EDL933 in *C. elegans* (Fig. 6c), suggesting that TnaA is also a virulence determinant of EHEC. We noted that the virulence of the EDL933:Δ*sdhA*Δ*tnaA* strain to *C. elegans* animals was more significantly decreased than that of the EDL933:Δ*tnaA* strain, but is similar to that of the EDL933:Δ*sdhA* mutant (Fig. 6d). These genetic epistasis results placed the *tnaA* downstream and in the same pathway of *sdhA*. Together with our proteomic and qRT-PCR analyses, these results demonstrated that the expressions of the TnaA protein and mRNA are *sdhA*-and fumarate*-*dependent.

### Cra is required for fumarate-dependent virulence regulation in EHEC

Fumarate and succinate belong to the C4-dicarboxylates that are metabolized by bacteria under aerobic or anaerobic conditions^32^. Sensing and uptaking of C4-dicarboxylates are achieved by several two-component systems in bacteria. We have tested three putative two-component systems for C4 dicarboxylates regulation, including the DcuSR, DctSR, and DctBD, and found there are not involved in sensing fumarate to regulate the virulence of EHEC (Supplemental Figure S5).

**Fig. 7:**
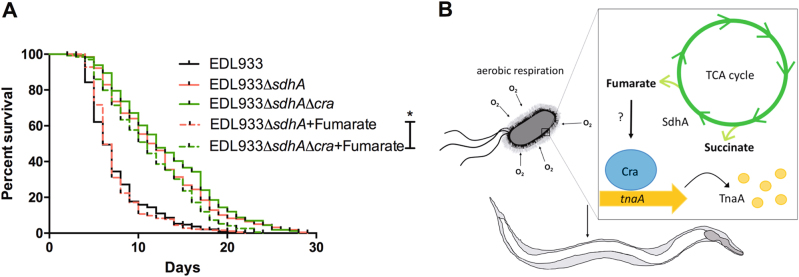
Fumarate regulates EHEC virulence through the Cra transcription factor. **a** The survival of N2 worms fed with the wild-type strain (EDL933) and the isogenic deletion strains of *sdhA* (EDL933:Δ*sdhA*), the *sdhA* and *cra* double mutant (EDL933:Δ*sdhA*Δ*cra*) and mutants treated with 2.5 mM fumarate, respectively (EDL933:Δ*sdhA* + Fumarate and Δ*sdhA*Δ*cra* + Fumarate) were examined. The virulence of *sdhA* and *cra* double mutant treat with 2.5 mM fumarate (EDL933:Δ*sdhA*Δ*cra* + Fumarate, median N2 lifespan = 10.3 ± 0.9 days) was similar to *sdhA* mutant treated with 2.5 mM fumarate (EDL933:Δ *sdhA* + Fumarate, median N2 lifespan = 6.3 ± 0.3 days, *P* < 0.0001). The asterisk denotes statistically significant (*P* < 0.0001) examined by the log-rank test and “ns” represents no significant difference statistically. **b** A schematic model illustrating the roles of aerobic metabolism (aerobic respiration and TCA cycle), succinate dehydrogenaae (SdhA), and especially the metabolite, fumarate, in controlling the virulence of EHEC through a TnaA- and Cra-dependent manner

Recently, a transcription factor Cra (FruR) which regulates carbohydrate metabolic enzymes to control sugar concentration in *E. coli* has been reported to modulate EHEC virulence effectors that are required for formation of A/E lesions^8,33^. Given that Cra has also been reported to function as a metabolic flux sensor in *E. coli*^34^, we tested whether Cra can sense fumarate and restore *sdhA* mutant toxicity. As shown in Fig. 7a, treatment of 2.5 mM fumarate can fully restore the virulence of the *sdhA* single mutant. However, this effect of fumarate was totally blocked in the *sdhAcra* double mutant, suggesting that fumarate rescue of the toxicity of *sdhA* mutant is Cra-dependent. Taken together, these findings show that fumarate regulates EHEC toxicity through the Cra transcription factor.

## Discussion

Succinate dehydrogenase (Sdh) complex is involved in both the TCA cycle and the aerobic respiratory chain simultaneously. Here, we demonstrated that disruption of the *sdhA* gene attenuated the toxicity of EHEC in vivo. Our data also showed that disruptions of the TCA cycle and aerobic metabolism both have effects on the toxicity attenuation of the *sdhA* mutant. Moreover, we demonstrated that down regulation of the virulence determinant *tnaA* in part accounts for the attenuated toxicity of the EHEC *sdhA* gene disruption. Interestingly, the attenuated pathogenicity of the *sdhA* mutant and the expression of *tnaA* in the *sdhA* mutant were significantly restored by supplementing with fumarate, the metabolite of SdhA, in a Cra-dependent manner. A model summarizing our findings is presented in the Fig. 7b. However, whether fumarate can directly or indirectly modulate Cra and how Cra can regulate the transcription of TnaA are warranted to test. Nevertheless, our results demonstrated that the bacterial metabolite fumarate played a major role in the pathogenesis of EHEC in vivo.

The TCA cycle has also been reported to be required for the virulence of the other enteric pathogens, *Salmonella enterica* serovar Typhimurium and uropathogenic *E. coli*^35–38^. Our results are in agreement with these reports, and we demonstrated, to our knowledge, for first time, the mechanism of the Sdh complex and mode of action of its catabolite, fumarate, in controlling EHEC virulence in vivo. Aforementioned that there is a relatively aerobic zone adjacent to the mucosal surface of the intestinal epithelial cells caused by diffusion of oxygen from the microvilli capillary network^7^. It has been reported that variable oxygen content also modulates EHEC virulence in a vertical diffusion chamber system^39^. In addition, aerobic respiratory metabolism has also been reported to contribute to EHEC colonization of mouse intestine^6,40^. Our results here are in accordance with these observations, we demonstrated that EHEC is more toxic upon aerobiosis and is less toxic upon anaerobiosis to *C. elegans*.

Multiple metabolites of central carbon metabolism have been reported to regulate *C. elegans* lifespan. For example α-ketoglutarate, malate, fumarate, oxaloacetate, and acetate can extend *C. elegans* lifespan^41–44^. On the other hand, glucose shortens the lifespan of *C. elegans*^45^. Moreover, some metabolites such as tryptophan, indole, glycogen, and maltose also modulate the virulence of pathogenic bacteria^3,4,46^. In our study, we demonstrated that fumarate supplement in the *sdhA* mutant could significantly reverse the attenuated toxicity of the *sdhA* mutant. However, there was no obvious effect on the toxicity when the wild-type *E. coli* EDL933 strain and OP50 strain were treated with fumarate. A recent report suggested that fumarate increases the lifespan of *C. elegans*^44^, while we did not observe the same effect in our studies. This discrepancy may be the result of the different culture conditions of *C. elegans* and the concentration of fumarate used in our study.

Interestingly, it has been suggested that oxygen levels varying in the gastrointestinal tract modulate intracellular enteric pathogen *Shigella flexneri* virulence^7^. The *fnr* gene deletion mutant reduces the *Shigella flexneri* l colonization in the intestine and reduces the destruction of the mucosal surface of villi. However, in our study, the EDL933 mutant with *fnr* gene deletion expressed similar toxicity as wild-type. These results suggested that the regulation of virulence by the switch between aerobic and anaerobic respirations is strain-specific and may be Fnr-independent in EHEC. Cra is a universal regulator that regulates genes involved in carbon metabolism and can also regulate EHEC virulence expression dependent on glucose concentration^8^. A recent report showed that metabolism and oxygen availability can affect EHEC virulence via Cra^47^. Here we demonstrated that EHEC is less toxic toward *C. elegans* under anaerobic culture and this regulation might be controlled by the metabolite, fumarate. Consistent with the idea that when EHEC shifts to the aerobic zone of gut epithelial cells in GI tract, EHEC activates T3SS expression enhancing its adherence to epithelial cells and this activation is dependent on Cra regulation^39,47^.

Pathogenic *E. coli* may require certain metabolites, nutrients or specific metabolic pathways different from commensal *E. coli* and these differences may contribute to the pathogenicity. On the other hand, pathogens may utilize alternative pathways or mechanisms to evade the cell toxic metabolites or metabolic pathways resulting in reducing its pathogenicity. We envision that such EHEC-specific metabolite or metabolic pathways could be the potential targets for anti-infection.

## Electronic supplementary material

Supplemental Information
