# Supplementary material for: A multi-omic analysis reveals the role of fumarate in regulating the virulence of enterohemorrhagic Escherichia coli
Source: Cell Death Dis. 2018 Mar 7;9(3):381. doi: 10.1038/s41419-018-0423-2 (PMC5841434; doi:10.1038/s41419-018-0423-2)
Supplement: Supplementary file 1 — Supplemental Information [file 41419_2018_423_MOESM1_ESM.pdf]

Supplemental Information

Experimental Procedures:

Measurement of bacterial growth curve

Bacterial cells were initially cultured in LB broth at 37°C for 16 hours. At the next day, the overnight cultures were adjusted to O.D.$_{600}$=1.2 and inoculated into 5 mL fresh LB broth with 1:500 dilution. The O.D.$_{600}$ values for the bacterial growth curve were automatically measured every 30 min using an OD-Monitor C&T (Taitec, Saitama, Japan) as described (1).

Construction of EHEC deletion mutants

The isogenic gene deletion mutants were constructed by the Lambda Red-mediated recombination system as described (2, 3). The Lambda Red recombinase expression plasmid pKD46 is a temperature-sensitive plasmid, and the lambda red proteins were induced with 10 mM L-arabinose. This method was performed using primers containing the sequence for 30 bp homology to the target gene and 20 bp to amplify a kanamycin or chloramphenicol resistance gene from pKD4 or pKD3. The primers used in the mutant construction are listed in Table S2. The antibiotic resistance genes were flanked by recombinase FLP recognition target (FRT) sites, and directly repeated FRT sites were used for antibiotic resistance gene removal with pCP20. For generation of the EHEC strain EDL933 deletion mutants, the purified DNA fragments were electroporated into EDL933 harboring pKD46 cells. After electroporation, cells were incubated with 2XYT at 37°C for 1 hour, and plated on an LB agar plate containing kanamycin or chloramphenicol. The plates were incubated at 37°C for antibiotic screening and to induce the loss of pKD46. In order to generate EDL933 multiple gene mutants or prevent the polar effects on upstream and downstream gene expression of target genes, it is necessary to remove the resistance cassette with pCP20. The Flp recombinase expression plasmid pCP20 is also a temperature-sensitive plasmid, and the expression of Flp recombinase is induced at 43°C (2-4). Flp recombinase recognizes the FRT sites and removes the FRT site-flanked antibiotic resistance gene, generating an in-frame deletion mutant. The selected colonies were sensitive to Ampicillin and Kanamycin or chloramphenicol for absence of pCP20 and the resistance gene.

Figures

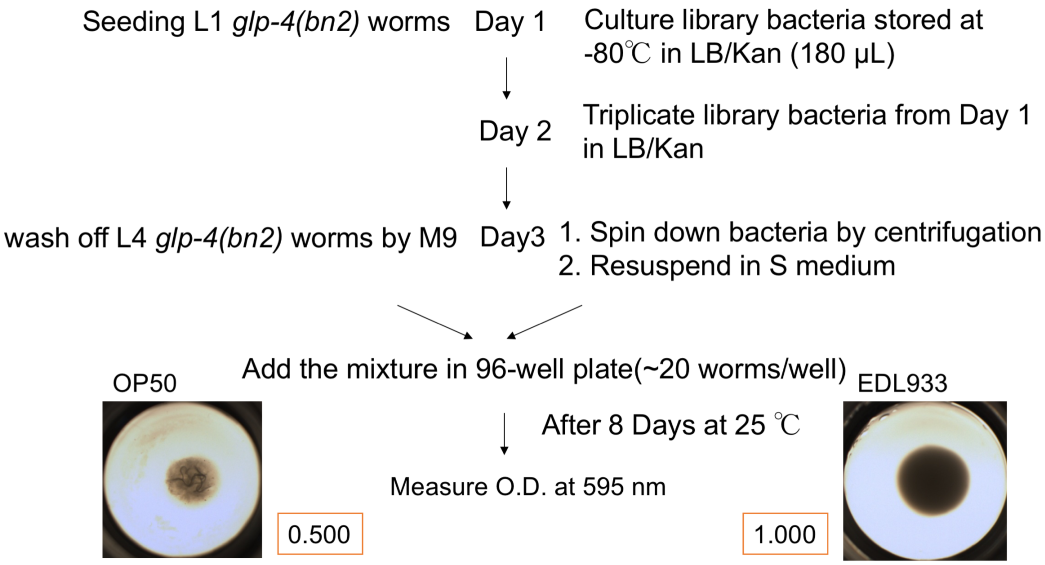

Figure S1. Screening of the EDL933 transposome mutant library.

C. elegans glp-4 (bn2) L1 stage larvae were cultured on the Enriched Nematode Growth (ENG) medium plates at the restrictive temperature (25°C) at Day 1. At the same day, the EDL933 transposome mutant library, stored in 96-well plates and in -80°C freezers, was replicated in LB broth containing 50 μg/mL Kanamycin (Kan) and put in a 37°C incubator for 16 to 18 hours. At Day 2, the entire library was triplicated in 96-well plates containing LB broth with 50 μg/mL Kan and cultured at 37 °C for another 16 to 18 hours. At Day 3, when *C. elegans glp-4 (bn2)* animals reached to L4 larvae/young adult stage, the worms were washed off from ENG plates by M9 buffer and collected. These worms were mixed with each transposon mutant clones in 96-well plated, which was centrifuged and resuspended in S medium. Each well contained approximately 20 worms. Then, the 96-well plates were placed at 25°C with shaking at 70 rpm. After 8 days, the $O.D._{595}$ values of each well were measured. The $O.D._{595}$ value was close to 0.5 when worms were cultured with *E. coli* strain OP50 (as negative control). In contrast, the $O.D._{595}$ value was around 1.0 when the worms were fed with EHEC wild-type EDL933 (as positive control). The hits/candidates with a decreased pathogenic phenotype toward *C. elegans* were selected with the O.D. value that was significantly lower compared to the EHEC wild-type EDL933 positive controls ($P<0.05$).

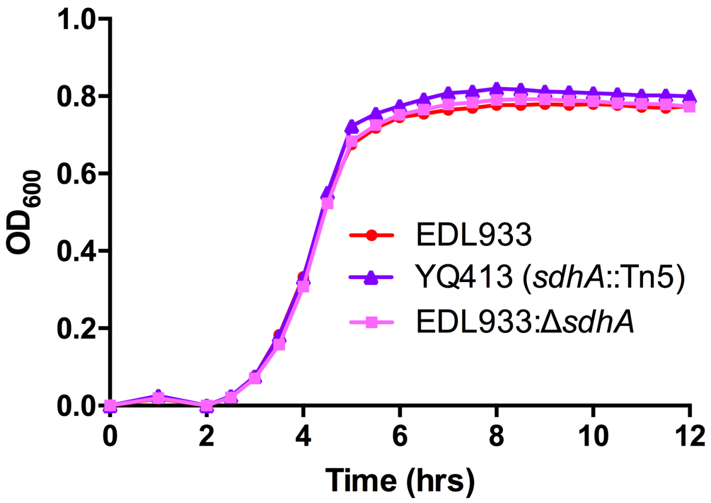

Figure S2. **Growth curves of the EHEC strains.**

The growth curves of the wild-type EHEC strain EDL933 (EDL933), the isogenic *sdhA* transposon mutant [YQ413 (*sdhA::*Tn*5*)], and the isogenic *sdhA* deletion mutant (EDL933*:*Δ*sdhA*) were measured.

Anaerobic metabolism is dispensable for the full virulence of EHEC in *C. elegans*

During anaerobic metabolism, the TCA cycle is repressed and nitrate catalyzed by nitrate reductase (Nar) and fumarate catalyzed by fumarate reductase (Frd) can both act as the alternative terminal electron acceptors other than oxygen (5); or alcohol dehydrogenase, encoded by the *adhE* gene, can regenerate NAD^+ for glycolysis and control fermentation in *E. coli* (6). Moreover, the transcriptional regulator Fnr (fumarate/nitrate reduction regulator) is required for anaerobic respiration and controls the switch from aerobic to anaerobic respiration (7), and the ribonucleotide reductase class III, encoded by *nrdD* and *nrdG*, is essential for a strictly anaerobic environment in *E. coli* (8). To test whether anaerobic metabolism, including anaerobic respiration and fermentation, also plays roles in the pathogenesis of EHEC in *C. elegans,* five isogenic mutants with *narHJI, frdA, adhE, fnr*, and *nrdDG* deletion (EDL933:Δ*narHJI*, EDL933:Δ*frdA*, EDL933:Δ*adhE*, EDL933:Δ*fnr,* and EDL933:Δ*nrdDG)* were generated and tested. We noted that these isogeneic mutants were as toxic as the parental wild-type EDL933 (Figure S3). Given the potential redundancy of these genes in controlling anaerobic metabolism, a compound mutant was also generated. Our results showed that the isogeneic EDL933:Δ*narHJI*Δ*frdA*Δ*adhE*Δ*fnr*Δ*nrdDG* mutant strain was as toxic as the wild-type EDL933 (Figure S3). Together, our current data suggested that anaerobic metabolism is dispensable for the full virulence of EHEC in *C. elegans*.

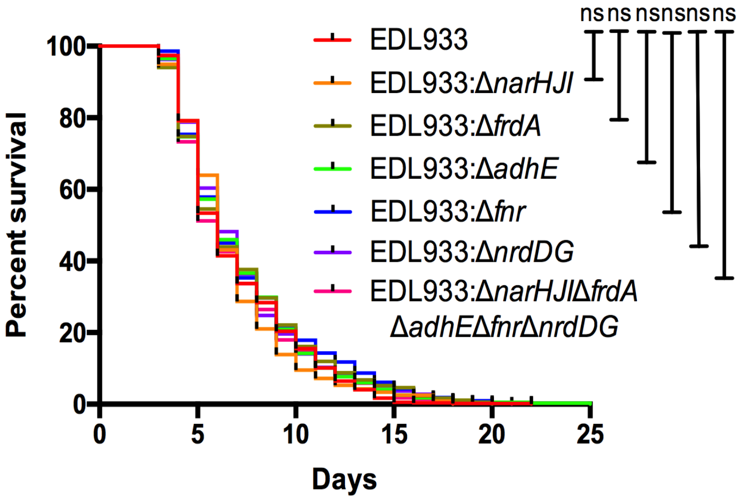

Figure S3. Deletion of genes involved in anaerobic metabolism did not alter EHEC toxicity in *C. elegans*.

The survival of N2 worms fed with the wild-type EDL933 (EDL933) and the isogenic deletion strains of *narHJI* (EDL933:Δ*narHJI*), *frdA* (EDL933:Δ*frdA*), *adhE* (EDL933:Δ*adhE*), *fnr* (EDL933:Δ*fnr*), and *nrdDG* (EDL933:Δ*nrdDG*) were examined. Deletion of *narHJI* (median N2 lifespan = 6.0 ± 0.1 days, *P*=0.205), *frdA* (median N2 lifespan = 6.7 ± 0.6 days, P=0.129), *adhE* (median N2 lifespan = 6.0 ± 0.1 days, *P*=0.413), *fnr* (median N2 lifespan = 6.0 ± 0.1 days, *P*=0.448), and *nrdDG* (median N2 lifespan = 6.5 ± 0.7 days, *P*=0.908) were as toxic as the parental wild-type EDL933 (median N2 lifespan = 6.2 ± 0.5 days). “ns“ represents no statistically significant difference examined by the Log-rank test.

The effect of fumarate is specific to EHEC

The survival curves of *C. elegans* animals did not change when fed on the succinate or fumarate treated OP50 (Figure S4A). These results suggested that the effect of fumarate was on EDL933:Δ*sdhA* mutant directly. We also generated the isogeneic *sdhA* mutant strain of *E. coli* OP50 (OP50:Δ*sdhA*) to examine whether the effect of fumarate is specific to EHEC. Our results showed that the survival curves of *C. elegans* animals fed on the wild-type OP50 and the OP50:Δ*sdhA* mutant were similar (Figure S4B). Moreover, the survival curves of *C. elegans* animals fed on succinate or fumarate treated OP50:Δ*sdhA* were similar to the untreated control, which suggested that the *sdhA* gene is specifically required for the pathogenesis of EHEC in *C. elegans*.

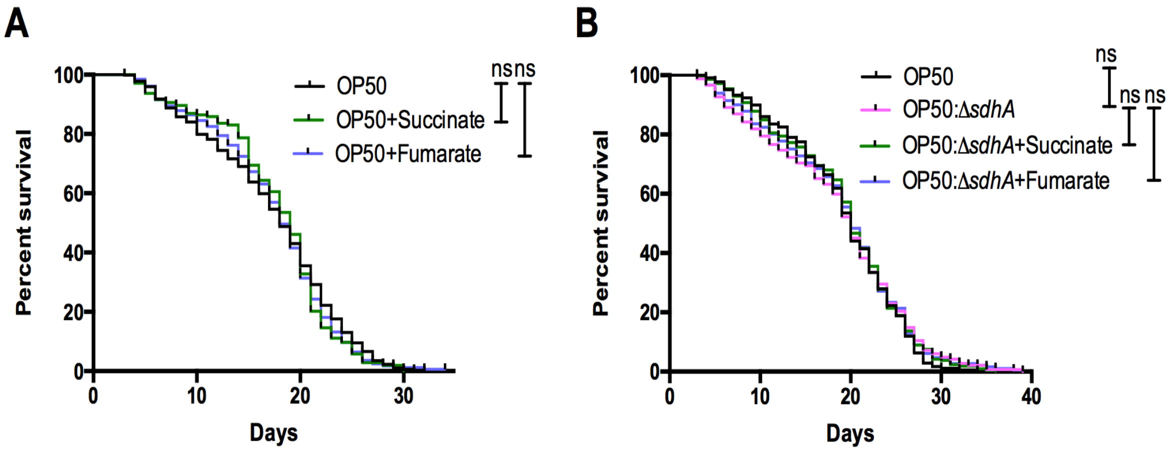

Figure S4. **Supplement of *E. coli* OP50 and OP50:Δ*sdhA* with succinate or fumarate did not alter *C. elegans* lifespan.**

(A) The survival curves of worms fed with the wild-type OP50 strain cultured with 2.5 mM succinate (OP50+Succinate) or fumarate (OP50+Fumarate) were examined. Animals on OP50 treated with succinate (OP50+Succinate, N2 median lifespan = 18.5 ± 1.5 days, *P*=0.72) or fumarate (OP50+Fumarate, N2 median lifespan = 17.8 ± 0.49 days, *P*=0.40) shown a similar lifespan compared to that on OP50 (OP50, N2 median lifespan = 18.67 ± 0.42 days). (B) The survival curves of worms fed with the wild-type OP50 strain, and OP50 with isogenic deletion strain of *sdhA* (OP50:Δ*sdhA*) cultured with 2.5 mM succinate (OP50:Δ*sdhA*+Succinate) or fumarate (OP50:Δ*sdhA*+Fumarate) were examined. Worms on the OP50:Δ*sdhA* strain (OP50:Δ*sdhA,* N2 median lifespan = 20.0 ± 1.4 days, *P*=0.627) exhibited similar lifespan compared to the wild-type OP50

strain (OP50, N2 median lifespan = 20.5 ± 0.7 days) toward *C. elegans* animals. Worms on succinate-treated OP50:Δ*sdhA* strain (OP50:Δ*sdhA*+Succinate*,* N2 median lifespan = 20.0 ± 0.1 days, *P*=0.842) and fumarate-treated OP50:Δ*sdhA* strain (OP50:Δ*sdhA*+Fumarate, N2 median lifespan =20.5 ± 0.7 days, *P*=0.878) all exhibited similar lifespan compared to the untreated control (OP50:Δ*sdhA,* N2 median lifespan = 20.0 ± 1.4 days). “ns“ represents no statistically significant difference examined by the Log-rank test.

The three putative C4-dicarboxylates sensor-regulator systems are dispensable

The *dcuSR* operon (also known as *yjdHG*) encodes a two-component sensor-regulator system (DcuS-DcuR) which can sense fumarate and lead to activation of the fumarate-succinate antiporter DcuB expression in *E. coli* (9, 10). If fumarate restores *sdhA* mutant toxicity/virulence through the DcuSR two-component system, deletion of *dcuSR* in the *sdhA* mutant background cannot restore its toxicity after supplement of fumarate. We therefore generated the *sdhAdcuSR* isogenic mutant and examined its toxicity to *C. elegans* under fumarate supplement. As shown in Figure S5A, the toxicity of *sdhAdcuSR* mutant to *C. elegans* was significantly attenuated compared with wild-type EHEC (P<0.0001) but was similar to the *sdhA* single mutant (P=0.151). Moreover, addition of 2.5 mM fumarate not only restored the toxicity of *sdhA* mutant but also the *sdhAdcuSR* mutant which suggested that the *dcuSR* two-component system is not involved in sensing fumarate to regulate the virulence of EHEC.

Another DctS-DctR two-component system, which encoded by *dctS* and *dctR* genes, is required for high-affinity C4-dicarboxylate transport in *Rhodobacter capsulatus* (9, 11). We blasted the amino acid sequence of DctS and DctR to the EDL933 amino acid sequence and identified YhiF (Z4909, *yhiF*) as a close homolog of DctR, but could not identify any homolog of DctS. The DctB-DctD sensor-regulator controls the expression of the *dctA* gene encoding C4-dicarboxylate transporter DctA in *Rhizobia* (11). We also blasted the amino acid sequence of DctB and DctD to EDL933 protein sequence and identified HyfR (Z3751, *hyfR*) as having the closest homology to DctD. However, we could not identify any DctB homolog in EDL933. Therefore, we generated the isogenic mutant of *dctR* (*yhiF)* and *dctD* (*hyfR)* in the *sdhA* mutant background to examine whether fumarate regulates EDL933 virulence through SdhA via these two-component systems. As shown in Figure S5B, *dctRsdhA* double mutant is less toxic to *C. elegans* compared with wild-type EHEC (P < 0.0001) but is similar to the *sdhA* single mutant (P=0.96). Supplement of 2.5 mM fumarate to the *dctRsdhA* double mutant restored its toxicity to that of the *sdhA* single mutant (P=0.57), suggesting that the DctS-DctR two-component sensing pathway is not required for fumarate to regulate EHEC toxicity.

We also generated *dctD* isogenic mutant in the *sdhA* mutant background and examined its toxicity toward *C. elegans* when supplied with 2.5 mM fumarate. In the same manner as the *dctRsdhA* double mutant, addition of fumarate to the *dctDsdhA* double mutant rescued its toxicity to that of the *sdhA* single mutant (P=0.86) (Figure S5C).

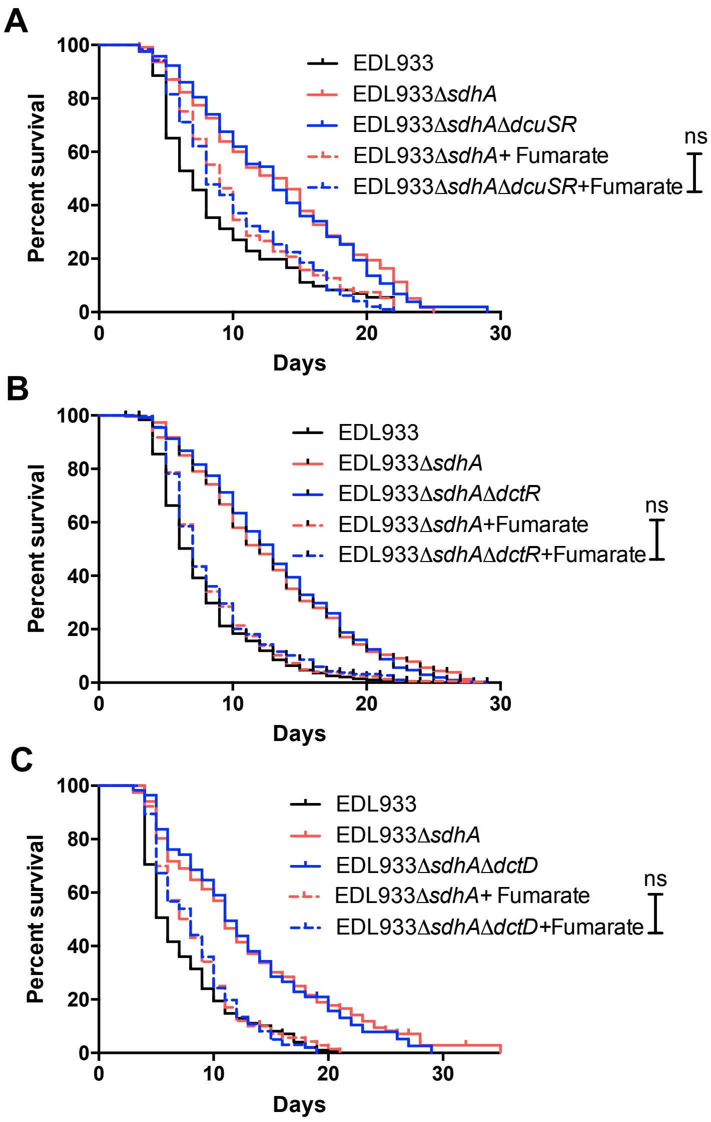

Figure S5. **Deletion of the putative two-component systems in C4 dicarboxylates regulation did not affect the capability of fumarate to restore the toxicity of the EHEC *sdhA* mutant.**

(A) The survival of N2 worms fed with the wild-type strain (EDL933) and the isogenic deletion strains of *sdhA* (EDL933:Δ*sdhA)*, the *sdhA* and *dcuSR* triple mutant (EDL933:Δ*sdhA*Δ*dcuSR*) and mutants treated with 2.5mM fumarate, respectively (EDL933:Δ*sdhA*+Fumarate and EDL933:Δ*sdhA*Δ*dcuSR*+Fumarate), were examined. The virulence of *sdhA* and *dcuSR* triple mutant treat with 2.5mM fumarate (EDL933:Δ*sdhA*Δ*dcuSR*+Fumarate, median N2 lifespan = 9 days) was similar to *sdhA* mutant treated with 2.5 mM fumarate (EDL933:Δ*sdhA*+Fumarate, median N2 lifespan = 8 days, *P*=0.52*)*. (B) The survival of N2 worms fed with the wild-type strain (EDL933)

and the isogenic deletion strains of *sdhA* (EDL933:Δ*sdhA)*, the *sdhA* and *dctR* double mutant (EDL933:Δ*sdhA*Δ*dctR*) and mutants treated with 2.5mM fumarate, respectively (EDL933:Δ*sdhA*+Fumarate and EDL933:Δ*sdhA*Δ*dctR*+Fumarate) were examined. The virulence of *sdhA* and *dctR* double mutant treated with 2.5 mM fumarate (EDL933:Δ*sdhA*Δ*dctR*+Fumarate, median N2 lifespan = 7.3 ± 0.6 days) was similar to *sdhA* mutant treated with 2.5mM fumarate (EDL933:Δ*sdhA*+Fumarate, median N2 lifespan = 7.4 ± 0.5 days, *P*=0.57*)*. (C) The survival of N2 worms fed with the wild-type strain (EDL933) and the isogenic deletion strains of *sdhA* (EDL933:Δ*sdhA)*, the *sdhA* and *dctD* double mutant (EDL933:Δ*sdhA*Δ*dctD*) and mutants treated with 2.5 mM fumarate, respectively (EDL933:Δ*sdhA*+Fumarate and EDL933:Δ*sdhA*Δ*dctD*+Fumarate), were examined. The virulence of *sdhA* and *dctD* double mutant treat with 2.5 mM fumarate (EDL933:Δ*sdhA*Δ*dctD*+Fumarate, median N2 lifespan = 8 days) was similar to *sdhA* mutant treated with 2.5 mM fumarate (EDL933:Δ*sdhA*+Fumarate, median N2 lifespan = 8 days, *P*=0.86*)*. “ns“ represents no statistically significant difference examined by the Log-rank test.

Tables

Table S1. Nematode strains used in this study.

Strain	Relevant characteristics	Source or reference
N2	*C. elegans* wild-type strain	(12)
GK454	*unc-119(ed3), dkIs247[Pact-5::mCherry::HA::act-5, unc119(+)];* mCherry::ACT-5 expression	(13)

Table S2. Bacterial strains used in this study.

Strain	Description	Source or reference
OP50	uracil auxotroph and laboratory food source for *C. elegans*	(12)
EDL933	*E. coli* O157:H7 isolated from raw hamburger meat	(14)
HER1266	*E. coli* O157:H7 isolated from human stool	(15)
YQ413	*sdhA::*Tn*5,* Tn*5* transposon mutant inserted in the *sdhA* gene of EDL933	this study
EDL933:Δ*sdhA*	EDL933 isogenic mutant with *sdhA* gene deleted; Kan^R kick out	this study
EDL933:Δ*sdhC*	EDL933 isogenic mutant with *sdhC* gene deleted; Kan^R	this study
EDL933:Δ*sdhD*	EDL933 isogenic mutant with *sdhD* gene deleted; Kan^R	this study
EDL933:Δ*sdhB*	EDL933 isogenic mutant with *sdhB* gene deleted; Kan^R	this study
EDL933:Δ*sdhC*Δ*sdhD* Δ*sdhA*Δ*sdhB*	EDL933 isogenic mutant with *sdhCDAB* operon deleted; Kan^R	this study
EDL933-pQE30	EDL933 transformed with pQE30; Amp^R	(16)
EDL933:Δ*sdhA*-pQE30	EDL933 isogenic mutant with sdhA gene deleted; Kan^R kick out, and transformed with pQE30; Amp^R	this study
EDL933:Δ*sdhA-pWF134*	EDL933 isogenic mutant with sdhA gene deleted; Kan^R kick out, and transformed with pWF134; Amp^R	this study
EDL933:Δ*sdhA-pWF134*	EDL933 isogenic mutant with *sdhA* gene deleted; Kan^R, and complement with *sdhCDAB* by transformation with pWF134; Amp^R	this study
EDL933:Δ*sdhCDAB-pWF134*	EDL933 isogenic mutant with *sdhCDAB*gene deleted; Kan^R, and complement with *sdhCDAB* by transformation with pWF134; Amp^R	this study
EDL933 *sdhA::*Tn*5 -pWF134*	EDL933 transposon inserted in *sdhA* gene; Kan^R, and complemented with *sdhCDAB* by transformation with pWF134; Amp^R	this study
OP50:Δ*sdhA*	OP50 isogenic mutant with *sdhA* gene deleted; Kan^R	this study

Strain	Description	Source or reference
EDL933:ΔicdA	EDL933 isogenic mutant with *icdA* gene deleted; Kan^R	this study
EDL933*:ΔsucAΔsucB*	EDL933 isogenic mutant with *sucA* gene and *sucB* gene deleted; Kan^R	this study
EDL933*:ΔsucCΔsucD*	EDL933 isogenic mutant with *sucC* gene and *sucD* gene deleted; Kan^R	this study
EDL933*:ΔfrdA*	EDL933 isogenic mutant with *frdA* gene deleted; Kan^R	this study
EDL933:Δ*fumC*Δ*fumA*	EDL933 isogenic mutant with *fumC* gene and *fumA* gene deleted; Kan^R	this study
EDL933:Δ*mdh*	EDL933 isogenic mutant with *mdh* gene deleted; Kan^R	this study
EDL933:Δ*gltA*	EDL933 isogenic mutant with *gltA* gene deleted; Kan^R	this study
EDL933:ΔygfH	EDL933 isogenic mutant with *ygfH* gene deleted; Cm^R	this study
EDL933:Δ*sdhA*Δ*ygfH*	EDL933 isogenic mutant with *sdhA* gene and *ygfH* gene deleted; Kan^R Cm^R kick out	this study
EDL933:Δ*arcA*	EDL933 isogenic mutant with *arcA* gene deleted; Kan^R	this study
EDL933:Δ*arcB*	EDL933 isogenic mutant with *arcB* gene deleted; Kan^R	this study
EDL933:Δ*arcA*Δ*arcB*	EDL933 isogenic mutant with *arcA* gene and *arcB* gene deleted; Kan^R Cm^R	this study
EDL933:Δ*fnr*	EDL933 isogenic mutant with *fnr* gene deleted; Kan^R	this study
EDL933:Δ*narH*Δ*narJ* Δ*narI*	EDL933 isogenic mutant with *narH* gene, *narJ* gene, and *narI* gene deleted; Kan^R	this study
EDL933:Δ*adhE*	EDL933 isogenic mutant with *adhE* gene deleted; Kan^R	this study
EDL933:ΔnrdDΔnrdG	EDL933 isogenic mutant with *nrdD* gene and *nrdG* gene deleted; Kan^R	this study
EDL933:Δ*narH*Δ*narJ* Δ*narI*Δ*fnr*Δ*adhE*	EDL933 isogenic mutant with *narHnarJnarI* gene deleted; Kan^R kick out , *fnr* gene; Kan^R, and *adhE* gene; Cm^R	this study
EDL933:Δ*sdhA*Δ*tnaA*	EDL933 isogenic mutant with *sdhA* gene; Kan^-, and *tnaA* gene deleted; Kan^R	this study

Table S3. Plasmids used in this study.

Plasmid	Relevant characteristics	Source or reference
pFPV25.1	Vector for constitutive GFP expression; *rpsM::gfpmut*; Amp^R	(17)
pKD46	Red recombinase expression; Amp^R	(2, 18)
pKD3	Template plasmid for Cm^R cassette	(2)
pKD4	Template plasmid for Kan^R cassette	(2)
pQE30	Amp^R, T5 expression vector	Qiagen, USA
pCP20	FLP recombinase expression; Amp^R Cm^R	(2)
pWF134	*sdhCDAB* expressing plasmid; Amp^R	this study

Table S4. Primers used in cloning, mutant construction, and qRT–PCR in this study.

Name	Oligonucleotides
**Primers used for cloning (5' to 3')**	
pQE30-*sdhCDAB* F5	ACATGCATGCTTAAGGTCTCCTTAGCGCC
pQE30-*sdhCDAB* R3	ACGCGTCGACGCCGCATCCGGCACTGGTTG
**Primers used for mutant construction (5' to 3')**	
sdhA F5	GGATTCGTTGTGGTGTGGGGTGTGTGATGAGTGTAGGCTGGAGCTGCTTC
sdhA R3	CATTTTCCTGTCTCCGCATTAGTAAGTACGCATATGAATATCCTCCTTAG
sdhA Up R3	TCATCACACACCCCACACCACAACGAATCC
Check *sdhA* F5	CTATCTGGAAGAAACATTCG
Check *sdhA* R3	AGGGTGTAATCCTGCATAC
sdhB F5	GTACTTACTAATGCGGAGACAGGAAAATGAGTGTAGGCTGGAGCTGCTTC
sdhB R3	TCTTATCAGGCCTACGGTTTACGCATTACGCATATGAATATCCTC CTTAG
sdhB Up R3	TCATTTTCCTGTCTCCGCATTAGTAAGTAC
Check *sdhB* F5	AGCATAACTTCTCGGTCTTC
Check *sdhB* R3	ATACTACCACGCACAGTGAT
sdhC F5	ATAAGAACAGCATGTGGGCGTTATTCATGAGTGTAGGCTGGAGCTGCTTC
sdhC R3	CTAATGCGGAGGCGTTGCTTACCATACGAGCATATGAATATCCTCCTTAG
sdhC Up R3	TCATGAATAACGCCCACATG CTGTTCTTAT
Check *sdhC* F5	CTAATAACTGTCCCGAATGA
Check *sdhC* R3	ATAAATCACGTAAACCACCA
sdhD F5	CTTTCACTTCTCGCAGGAGTCCTCGTATGGGTGTAGGCTGGAGCTGCTTC
sdhD R3	CTCTGACTGGCAATTTCATCACACACCCCACATATGAATATCCTCCTTAG
sdhD Up R3	CCATACGAGGACTCCTGCGAGAAGTGAAAG
Check *sdhD* F5	TATCACGTCGTCGTAGGTAT
Check *sdhD* R3	CCGGTTTTACACATATATTC A
acnB F5	GAATACCGTAAGCACGTAGCTGAGCGTGCCGTGTAGGCTGGAGCTGCTTC
acnB R3	AGTCTGGAAAATCACCCCATCGGCTTTCTC CATAT GAATA TCCTC CTTAG
acnB Up R3	GGCACGCTCAGCTACGTGCTTACGGTATTC
Check *acnB* F5	TTCATAATTCGGATCTCAAG
Check *acnB* R3	TTCGTCGTAGTAGTTCATCC
icdA F5	AAAGTAGTTGTTCCGGCACAAGGCAAGAAGGTGTAGGCTGGAGCTGCTTC
icdA R3	CTTGATGATCGCGTCACCAAACTCTGAACACATATGAATATCCTCCTTAG
icdA Up R3	CTTCTTGCCTTGTGCCGGAACAACTACTTT
Check *icdA* F5	TATTGGTCAGCACCAGTAAC
Check *icdA* R3	CATTACCGTCACACTACCTC

sucA F5	AGCGCTTTGAAAGCCTGGTTGGACTCTTCTGTGTAGGCTGGAGCTGCTTC
sucB R3	CAGCAGCAGACGCGTCGGATCTTCCAGCAACATATGAATATCCTCCTTAG
sucA Up R3	AGAAGAGTCCAACCAGGCTTTCAAAGCGCT
Check *sucA* F5	AGTGTATTCCGCTGTCATAG
Check *sucB* R3	ACGTGAACTACGGTCTACAA
sucC F5	CGATTACTGAAGGATGGACAGAACACATGAGTGTAGGCTGGAGCTGCTTC
sucC R3	TATCAATTAAAATGGACATTATTTCCCCTCCATATGAATATCCTCCTTAG
sucC Down F5	GAGGGGAAATAATGTCCATTTTAATTGATA
Check *sucC* F5	TGGTAACGATCAAAGAGTTG
Check *sucC* R3	GGTGATAATCAGTTTGATGC
sucD F5	GTTGTTGCCGCAGTGGAGGGGAAATAATGTGTGTAGGCTGGAGCTGCTTC
sucD R3	ATTTCTTATTACAGATATTTATTTCAGAACCATATGAATATCCTCCTTAG
sucD Down F5	GTTCTGAAATAAATATCTGTAATAAGAAAT
Check *sucD* F5	GATCTGATTTGCCTCGAC
Check *sucD* R3	ATCCCTCTAAGAATTTTTGC
frdA F5	GGATAAAAACAATCTGGAGGAATGTCGTGCGTGTAGGCTGGAGCTGCTTC
frdA R3	ATTTTCAGGTTTTTCATCTCAGCCATTCGCCATATGAATATCCTCCTTAG
frdA Up R3	GCACGACATTCCTCCAGATTGTTTTTATCC
Check *frdA* F5	TCTCGTCAAATTTCAGACTT
Check *frdA* R3	GGGTCTGGATGTTAGTACC
fumC F5	AATTAATCAGGTGAGGAGTAGGCCATGAGTGTAGGCTGGAGCTGCTTC
fumC R3	GCACCTGTATGTTGCAGATTAACGCCCGGCCATATGAATATCCTCCTTAG
fumC Down F5	GCCGGGCGTTAATCTGCAACATACAGGTGC
Check *fumC* F5	TTTTACATGGCACGAAAG
Check *fumC* R3	TGGTTGGGCTAATAAACATA
fumA F5	CAAACCAGGCAGTAAGTGAGAAAACAATGTGTGTAGGCTG GAGCTGCTTC
fumA R3	CCCGAAGGGCGGCTCTGTTTATTTCACACACATATGAATATCCTCCTTAG
fumA Up R3	ACATTGTTTTCTCACTTACTGCCTGGTTTG
Check *fumA* F5	GATGAACCTGAATGGAGAGT
Check *fumA* R3	CTGTTTTGCTTTCGTTAAGT
mdh F5	TTTATCAATATAATAAGGAGTTTAGGATGAGTGTAGGCTGGAGCTGCTTC
mdh R3	TTATTATCCGCTAATCAATTACTTATTAACCATATGAATATCCTCCTTAG
mdh Down F5	GTTAATAAGTAATTGATTAGCGGATAATAA
Check *mdh* F5	TGAAGAAGGCTGAAATAATG
Check *mdh* R3	AACTGATGGGCATTAACAC
gltA F5	GCAATAAGGCGCTAAGGAGACCTTAAATGGGTGTAGGCTGGAGCTGCTTC
gltA R3	ATGGTTCAAATCAGATAATTAATGTTTAACCATATGAATATCCTCCTTAG
gltA Up R3	CCATTTAAGGTCTCCTTAGCGCCTTATTGC
check *gltA* F5	TCATTCGGGACAGTTATTAG

check *gltA* R3	CTTCATGGGCTATGATAAAG
ygfH F5	CAGTGGACAAGGATGACCGCCGATGAAGCGGTGTAGGCTGGAGCTGCTTC
ygfH R3	CATCGAGCCGGTTGCAATTAAATTACGGTGCATATGAATATCCTCCTTAG
ygfH Up R3	CGCTTCATCGGCGGTCATCCTTGTCCACTG
Check *ygfH* F5	TCAAAGAGCTGATTTTTACC
Check *ygfH* R3	CTTTTTGACCGTCAGTTAGA
arcA F5	TTTAGTTGGCAATTTAGGTAGCAAACATGCGTGTAGGCTGGAGCTGCTTC
arcA R3	TGACGGTGGTAAAGCCGATTAATCTTCCAGCATATGAATATCCTCCTTAG
arcA Up R3	GCATGTTTGCTACCTAAATTGCCAACTAAA
Check *arcA* F5	CCTGACTGTACTAACGGTTTA
Check *arcA* R3	TTCTGAACATACCGGTTTTA
arcB F5	GCAGGTTGTCGTGAAGGAATTCCCTAATGAGTGTAGGCTGGAGCTGCTTC
arcB R3	ACCCCGGTCTAGCCGGGGTCATTTTTAGTCATATGAATATCCTCCTTAG
arcB Up R3	TCATTAGGGAATTCCTTCACGACAACCTGC
Check *arcB* F5	CTGAAGGTGTGTTCTCACTTA
Check *arcB* R3	TGCGTGAAATAGCTAACAA
fnr F5	ATATCAATTACGGCTTGAGCAGACCTATGAGTGTAGGCTGGAGCTGCTTC
fnr R3	GTGAGTTATGCGGAAAAATCAGGCAACGTTCATATGAATATCCTCCTTAG
fnr Up R3	TCATAGGTCTGCTCAAGCCGTAATTGATAT
Check *fnr* F5	TGGAAAACACTACGCACTAT
Check *fnr* R3	TTATGCCAGACCACTTTAAT
narH F5	AATGATCAGGTACAGGAGAGCGTAAAATGAGTGTAGGCTGGAGCTGCTTC
narI R3	ATGTGAACTAAAATTCGCTTAGTGACGAGCCATATGAATATCCTCCTTAG
narH Up R3	TCATTTTACGCTCTCCTGTACCTGATCATT
Check *narH* F5	GGTATCCACTCCACCTACA
Check *narI* R3	CAAACGAATCCGTAATTAAA
adhE F5	AAGTTTAACATTATCAGGAGAGCATTATGGGTGTAGGCTGGAGCTGCTTC
adhE R3	GCCAGACAGCGCTACTGATTAAGCGGATTTCATATGAATATCCTCCTTAG
adhE Up R3	CCATAATGCTCTCCTGATAATGTTAAACTT
Check *adhE* F5	AGCCACCAAATCATACTACA
Check *adhE* R3	AAAAACCATCTGTTTTTGTG
nrdD F5	CATGTGATGAAACGAGACGGCTGCAAAGTGGTGTAGGCTGGAGCTGCTTC
nrdG R3	ATGATGCACCACCTGATTGCTGCTGCCGCGCATATGAATATCCTCCTTAG
nrdD Up R3	CACTTTGCAGCCGTCTCGTTTCATCACATG
Check *nrdD* F5	TTGTGATGCATAACTACGAA
Check *nrdG* R3	CAATTTAAAAGTGGTCGAAA
tnaA F5	TATGTAATGGAAAACTTTAAACATCTCCCTGTGTAGGCTGGAGCTGCTTC<br>TATGTAATGGAAAACTTTAAACATCTCCCTGTGTAGGCTGGAGCTGCTTC
tnaA R3	TTTCAGTTTTGCGGTGAAGTGACGCAATACCATATGAATATCCTCCTTAG

tnaA Up R3	AGGGAGATGTTTAAAGTTTTCCATTACATA
Check *tnaA* F5	TCTCATAAACACAGCCAATA
Check *tnaA* R3	ATACGTGGATTAGCGTGATA
**Primers used for real time RT-PCR (5' to 3')**	
qPCR *tnaA* F5	AGGGATTAGAACGCGGTATTG
qPCR *tnaA* R3	CGGAGTTACTGGTGATGGTTG
qPCR *dnaJ* F5	ACCAAAGAGATCCGCATTCC
qPCR *dnaJ* R3	ACGGCAAAGAAACCCTGG
qPCR *rpoA* F5	GTGACCCTTGAGCCTTTAGAG
qPCR *rpoA* R3	ACACCATCAATCTCAACCTCG

Table S5. Proteins with differential expression in the wild-type EHEC strain (EDL933), the isogenic *sdhA* deletion mutant (EDL933:Δ*sdhA*), and the *sdhA* gene complementation strain (EDL933:Δ*sdhA*-pWF134).

Protein Name	Mass(Da)	EDL933	Δ*sdhA*	*t*-Test	Fold Change
**Down regulation in Δ*sdhA* VS. EDL933**					
Agmatinase	33557	0.84 ± 0.13	0 ± 0	0.0032	-100
Alcohol dehydrogenase YqhD	42097	0.84 ± 0.14	0 ± 0	0.0033	-100
Aspartate--ammonia ligase	36691	1.11 ± 0.12	0 ± 0	0.0008	-100
Chaperone protein DnaJ	41044	1.47 ± 0.32	0 ± 0	0.0103	-100
Dimethyl sulfoxide reductase DmsA	90399	1.11 ± 0.29	0 ± 0	0.0193	-100
Flavodoxin-1	19737	1.39 ± 0.05	0 ± 0	1E-05	-100
GDP-L-fucose synthase	36141	0.63 ± 0.02	0 ± 0	0	-100
Hydrogenase-1 large chain	66253	1.18 ± 0.17	0 ± 0	0.0021	-100
Nitrate/nitrite response regulator protein NarL	23927	0.77 ± 0.15	0 ± 0	0.0075	-100
Periplasmic nitrate reductase	93130	0.7 ± 0.06	0 ± 0	0.0003	-100
Uncharacterized protein YibN	15596	0.7 ± 0.07	0 ± 0	0.0004	-100
Uncharacterized protein YniA	32474	0.84 ± 0.02	0 ± 0	0	-100
Succinate dehydrogenase iron-sulfur subunit	26770	4.06 ± 0.37	0.39 ± 0.39	0.0024	-10.31
Succinate dehydrogenase flavoprotein subunit	64422	9.5 ± 0.23	1.01 ± 1.01	0.0011	-9.45
Xaa-Pro aminopeptidase	49815	1.18 ± 0.13	0.22 ± 0.22	0.0179	-5.49
Universal stress protein E	35707	2.22 ± 0.36	0.52 ± 0.27	0.0190	-4.24
Osmotically-inducible protein Y	21074	1.39 ± 0.17	0.43 ± 0.22	0.0246	-3.22

Protein HemY	45245	1.82 ± 0.09	0.81 ± 0.14	0.0034	-2.24
Chaperone protein skp	17688	2.45 ± 0.19	1.21 ± 0.36	0.0392	-2.03
Fumarate reductase iron-sulfur subunit	27123	2.24 ± 0.3	1.19 ± 0.17	0.0370	-1.88
30S ribosomal protein S12	13737	4.41 ± 0.67	2.38 ± 0.12	0.0404	-1.86
Protein YdgH	33903	2.59 ± 0.26	1.4 ± 0.3	0.0389	-1.85
Tryptophanase	52773	37.08 ± 2.43	20.99 ± 2.55	0.0102	-1.77
Cystine-binding periplasmic protein	29039	1.54 ± 0.18	0.89 ± 0.03	0.0247	-1.72
3-mercaptopyruvate sulfurtransferase	30826	1.54 ± 0.06	0.9 ± 0.15	0.0186	-1.71
50S ribosomal protein L13	16019	9.35 ± 0.4	7.87 ± 0.28	0.0374	-1.19
Molecular chaperone Hsp31 and glyoxalase 3	31220	1.05 ± 0.02	0.89 ± 0.03	0.0080	-1.17
**Up regulation in Δ*sdhA* VS. EDL933**					
Inosine-5'-monophosphate dehydrogenase	52022	4.68 ± 0.07	5.41 ± 0.13	0.0071	1.16
Aconitate hydratase 2	93498	21.29 ± 0.24	24.85 ± 0.28	0.0006	1.17
2-oxoglutarate dehydrogenase E1 component	105062	20.31 ± 0.41	25.38 ± 1.66	0.0409	1.25
Phosphopentomutase	44370	6.21 ± 0.1	7.92 ± 0.49	0.0271	1.27
Phosphate acetyltransferase	77172	9.57 ± 0.13	12.38 ± 0.91	0.0383	1.29
Long-chain fatty acid transport protein	48539	2.11 ± 0.46	3.95 ± 0.37	0.0361	1.87
Putative uncharacterized protein	29257	1.54 ± 0.18	3.11 ± 0.06	0.0011	2.03
Flagellin	51295	5.09 ± 0.54	10.98 ± 1.25	0.0123	2.16
Flagellin (Fragment)	56672	3.38 ± 1.48	7.77 ± 0.54	0.0497	2.3
50S ribosomal protein L20	13497	0.21 ± 0.21	2.3 ± 0.43	0.0121	11.11
Phosphomannomutase	50340	0 ± 0	1.25 ± 0.37	0.0277	100

**Up regulation in Δ*sdhA-pWF134* vs. Δ*sdhA***					
Ampicillin resistance protein	31557	0 ± 0	1.23 ± 0.14	0.0008	100
Beta-galactosidase	116462	0 ± 0	2.14 ± 0.49	0.0120	100
Beta-lactamase	31515	0 ± 0	0.87 ± 0.12	0.0018	100
Beta-lactamase TEM	31515	0 ± 0	8 ± 0.38	3E-05	100
Chaperone protein DnaJ	41044	0 ± 0	1.51 ± 0.16	0.0007	100
Protein dcrB	19787	0 ± 0	1.29 ± 0.41	0.0347	100
Putative acyl-CoA thioester hydrolase ybhC	46082	0 ± 0	0.79 ± 0.04	5E-05	100
Succinate dehydrogenase	26784	0 ± 0	6.18 ± 1.21	0.0069	100
Succinate dehydrogenase flavoprotein subunit	64422	1.01 ± 1.01	71.48 ± 16.92	0.0141	70.92
Succinate dehydrogenase iron-sulfur subunit	26770	0.39 ± 0.39	26 ± 1.98	0.0002	65.79
Lactaldehyde dehydrogenase	52273	1.03 ± 0.62	2.96 ± 0.18	0.0414	2.86
Tryptophanase	52773	20.99 ± 2.55	47.72 ± 4.72	0.0075	2.27
Acriflavine resistance protein A	42197	2.22 ± 0.08	3.75 ± 0.39	0.0186	1.69
Adenylosuccinate synthetase	47345	6.33 ± 0.82	10.66 ± 0.72	0.0163	1.68
Chaperone protein DnaK	69115	27.4 ± 2.12	38.29 ± 2.53	0.0300	1.4
Chaperone protein HtpG	71449	14.15 ± 0.23	19.75 ± 1.98	0.0482	1.4
Protein GrpE	21741	2.3 ± 0.08	2.83 ± 0.09	0.0128	1.23
**Down regulation in Δ*sdhA-pWF134* VS. Δ*sdhA***					
Pyruvate dehydrogenase E1 component	99668	26.62 ± 0.08	23.11 ± 0.94	0.0203	-1.15
Aerobic respiration control protein ArcA	27292	4.31 ± 0.21	3.16 ± 0.31	0.0390	-1.36

DNA-directed RNA polymerase subunit beta	150632	37.4 ± 2.31	27.38 ± 2.2	0.0349	-1.37
Peroxiredoxin OsmC	15088	2.3 ± 0.08	1.66 ± 0.14	0.0185	-1.38
Transcriptional regulatory protein OmpR	27354	3.32 ± 0.39	2.17 ± 0.09	0.0454	-1.53
2-oxoglutarate dehydrogenase E1 component	105062	25.38 ± 1.66	16.31 ± 0.3	0.0057	-1.56
Uncharacterized protein YggE	26635	1.48 ± 0.16	0.94 ± 0.05	0.0323	-1.57
Aconitate hydratase 1	97677	14.08 ± 0.66	8.63 ± 1.54	0.0312	-1.63
Glutamate decarboxylase alpha	52699	98.17 ± 8.26	52.19±12.15	0.0352	-1.88
Mannose-1-phosphate guanylyltransferase 2	54270	3.05 ± 0.22	1.5 ± 0.28	0.0122	-2.04
Long-chain fatty acid transport protein	48539	3.95 ± 0.37	1.88 ± 0.34	0.0148	-2.1
Biosynthetic arginine decarboxylase	73886	2.43 ± 0.33	1.07 ± 0.29	0.0361	-2.28
Flagellin	51295	10.98 ± 1.25	2.81 ± 0.73	0.0048	-3.91
Probable phospholipid-binding protein MlaC	23963	2.45 ± 0.39	0.61 ± 0.34	0.0231	-4.04
Glutaminase 1	32844	0.88 ± 0.11	0.21 ± 0.21	0.0482	-4.15
HTH-type transcriptional regulator IscR	17337	1.12 ± 0.16	0.21 ± 0.21	0.0272	-5.27
Flagellin (Fragment)	56672	7.77 ± 0.54	1.36 ± 0.24	0.0004	-5.69
Cyclopropane-fatty-acyl-phospholipid synthase	43777.81	1.12 ± 0.06	0 ± 0	0	-100
Lysine-arginine-ornithine-binding periplasmic protein	27992	1.33 ± 0.23	0 ± 0	0.0044	-100
Protein phosphatase CheZ	23976	1.9 ± 0.5	0 ± 0	0.0184	-100

Table S6: Transposon candidate genes list

GO category	Total gene/hit number	Gene Name	Segment Type	Primary Hit	*P* value of liquid-based survival (EDL933 vs mutants)	*P* value of agar-based survival (EDL933 vs mutants)	Product or Function
Metabolism	26/32	*sdhA*	backbone	ED97-A-1	< 0.0001	< 0.0001	succinate dehydrogenase, flavoprotein subunite;Energy metabolism, carbon: TCA cycle
		sdhC	backbone	ED201-E-9	< 0.0001	< 0.0001	succinate dehydrogenase, cytochrome b556; Energy metabolism, carbon: TCA cycle
		gltA/sdhC	backbone	ED29-D-11, ED54-D-6	< 0.0001(ED54D-6)	< 0.0001 (ED29-D-11), < 0.0001 (ED54-D-6)	type II citrate synthase / succinate dehydrogenase, cytochrome b556; Energy metabolism, carbon: TCA cycle
		mdh	backbone	ED61-A-7, ED61-C-11	< 0.0001, < 0.0001	growth defect	malate dehydrogenase; Energy metabolism, carbon: TCA cycle
		manB	84 O-Island	ED56-G-9	< 0.0001	< 0.0001	phosphomannomutase; Central intermediary metabolism
		treC	backbone	ED38-B-9	< 0.0001	0.4107	trehalase 6-P hydrolase; Degradation of small molecules: Carbon compounds
		bioH	backbone	ED185-D-2	0.0073	< 0.0001	biotin biosynthesis; Biosynthesis of cofactors, carriers: Biotin
		fbp	backbone	ED1-A-7	< 0.0001	0.0929	fructose-bisphosphatase; Central intermediary metabolism: Gluconeogenesis
		nuoI	backbone	ED135-E-12	< 0.0001	0.7054	NADH dehydrogenase I chain I; Energy metabolism, carbon: Aerobic respiration
		nuoB	backbone	ED208-B-9	< 0.0001	0.0232	NADH dehydrogenase I chain B; Energy metabolism, carbon: Aerobic respiration
		nuoH	backbone	ED196-G-6	< 0.0001	< 0.0001	NADH dehydrogenase I chain H; Energy metabolism, carbon: Aerobic respiration
		nuoM	backbone	ED207-F-4	< 0.0001	0.2743	NADH dehydrogenase I chain M; Energy metabolism, carbon: Aerobic respiration
		nuoG	backbone	ED203-B-3	< 0.0001	0.0294	NADH dehydrogenase I chain G; Energy metabolism, carbon: Aerobic respiration
		atpI	backbone	ED86-H-8	< 0.0001	growth defect	membrane-bound ATP synthase; ATP-proton motive force interconversion
		atpD	backbone	ED30-F-7	< 0.0001	growth defect	membrane-bound ATP synthase, F1 sector, beta-subunit; ATP-proton motive force interconversion
		aroA	backbone	ED1-F-12	< 0.0001	0.003	5-enolpyruvylshikimate-3-phosphate synthetase; Amino acid biosynthesis: Chorismate
		thrB	backbone	ED139-D-5	< 0.0001	auxotroph	homoserine kinase; Amino acid biosynthesis: Threonine
		pfs	backbone	ED127-G-8	< 0.0001	0.0002	orf, hypothetical protein; Unknown function, 5'-methylthioadenosine/S-adenosylhomocysteine nucleosidase
		udhA	backbone	ED138-B-4, ED205-G-12, ED52-B-6	< 0.0001, < 0.0001, < 0.0001	0.0370 (ED138-B-4), 0.0468 (ED205-G-12),	putative oxidoreductase; Not classified
		ubiE	backbone	ED137-C-6	< 0.0001	growth defect	Biosynthesis of cofactors, carriers: Menaquinone, ubiquinone
		epd	Backbone	ED160-C-11	< 0.0001	0.5628	D-erythrose 4-phosphate dehydrogenase; Central intermediary metabolism
		gnd	backbone	ED143-B-7, ED184-D-5	< 0.0001, < 0.0001	< 0.0001(ED143-B-7) < 0.0001(ED184-D-5)	gluconate-6-phosphate dehydrogenase, decarboxylating
		pnp	backbone	ED52-F-4	< 0.0001	< 0.0001	polynucleotide phosphorylase; cytidylate kinase activity; Macromolecule synthesis, modification: RNA synthesis, modification, DNA transcription
		pta	backbone	ED194-E-2	< 0.0001	0.1578	phosphotransacetylase; Degradation of small molecules: Carbon compounds
		guaA	backbone	ED86-A-10, ED174-A-9	< 0.0001, < 0.0001	auxotroph	GMP synthetase (glutamine-hydrolyzing);Nucleotide biosynthesis: Purine ribonucleotide biosynthesis
		bioC	backbone	ED134 E-11	< 0.0001	< 0.0001	biotin biosynthesis; reaction prior to pimeloyl CoA
Lipopolysaccharide		*rfaD*	145 O-Island	ED46-C-7	< 0.0001	< 0.0001	ADP-L-glycero-D-mannoheptose-6-epimerase;Cell exterior constituents: Surface polysaccharides and antigens
		rfaG	Hypervariable	ED55-A-3, ED132-A-10, ED189-H-9, D177-D-12	< 0.0001, < 0.0001, < 0.0001, < 0.0001	< 0.0001 (ED55-A-3)	glucosyltransferase I; lipopolysaccharide core biosynthesis
		rfaC	Junction	ED122-G-3, ED203-A-4	< 0.0001, < 0.0001	< 0.0001, < 0.0001	heptosyl transferase I; lipopolysaccharide core biosynthesis
		rfaF	backbone	ED74-D-7	< 0.0001	< 0.0001	ADP-heptose--lps heptosyltransferase II; lipopolysaccharide core biosynthesis
		Z4405	backbone	ED196-F-4	< 0.0001	< 0.0001	putative kinase; Not classified, RfaE-like
		waaI	145 O-Island	ED48-B-1, ED84-G-10, ED58-D-3, ED51-A-6	0.0003 (ED84G-10), < 0.0001 (ED58D-3),	< 0.0001 (ED48-B-1), < 0.0001 (ED84-G-10)	putative LPS biosynthesis enzyme; Cell exterior constituents: Surface polysaccharides and antigens
		waaP	Junction	ED82-D-12,ED62-G-1,ED8-H-7,ED45-C-10	< 0.0001, < 0.0001, < 0.0001, < 0.0001	< 0.0001 (ED82-D-12), 0.2475(ED62-G-1)	putative LPS biosynthesis enzyme; Cell exterior constituents: Surface polysaccharides and antigens

& Cell exterior constituents biosynthesis	15/33	*wzy*	84 O-Island	ED156-G-2,ED62-G-3,ED185-E-2	< 0.0001, < 0.0001, 0.0005	0.0255 (ED156-G-2), 0.5712 (ED62-G-3)	O antigen polymerase; Cell exterior constituents: Surface polysaccharides and antigens
		manC	84 O-Island	ED190-D-5,ED137-E-4	< 0.0001, < 0.0001	< 0.0001 (ED137-E-4)	mannose-1-P guanosyltransferase; Cell exterior constituents: Surface polysaccharides and antigens
		per	84 O-Island	ED186-D-7,ED206-H-5,ED208-F-11	< 0.0001, 0.0009, < 0.0001	< 0.0001 (ED186-D-7)	perosamine synthetase; Cell exterior constituents: Surface polysaccharides and antigens
		wbdP	84 O-Island	ED68-E-1,ED130-A-11,ED105-H-5	< 0.0001, < 0.0001, < 0.0001	< 0.0001 (ED68-E-1)	glycosyl transferase; Cell exterior constituents: Surface polysaccharides and antigens
		csgB	backbone	ED210-H-9	< 0.0001	0.018	minor curlin subunit precursor, similar ro CsgA; Cell exterior constituents: Surface structures
		fcI	84 O-Island	ED134-E-4	< 0.0001	< 0.0001	fucose synthetase; Cell exterior constituents: Surface polysaccharides and antigens
		Z3198	84 O-Island	ED12-E-8, ED151-H-5	< 0.0001, < 0.0001	< 0.0001 (ED12-E-8), 0.0002(ED151-H-5)	GDP-mannose dehydratase; Cell exterior constituents: Surface polysaccharides and antigens
		wbdR	84 O-Island	ED155-D-11	< 0.0001	0.0032	acetyl transferase; Cell exterior constituents: Surface polysaccharides and antigens
Type three secretion system	4/4	*eae*	148 O-Island	ED184-F-2	< 0.0001	< 0.0001	intimin adherence protein; Extracellular functions: Secreted proteins
		Z2240/Z2241	62 O-Island/Junction	ED52-B-8	< 0.0001	0.5175	hypothetical protein/hypothetical protein, putative type III effector protein, T3SS effector-like protein EspR-homolog
		Z3919/Z3920	108 O-Island	ED38-H-5	0.0014	0.002	hypothetical protein/hypothetical protein, non-LEE-encoded type III effector, T3SS secreted effector EspW-like protein
		ler	148 O-Island	ED7-C-4	0.0005	0.6197	orf, hypothetical protein; Unknown function; DNA-binding protein H-NS, transcriptional regulator Ler-like
Two component system	2/3	*ompR*	backbone	ED14-C-5, ED176-A-4	< 0.0001, < 0.0001	< 0.0001	response regulator (sensor, EnvZ) affecting transcription of ompC and ompF: outer membrane protein synthesis; Global regulatory functions
		Z3603	backbone	ED4-B-11	< 0.0001	0.9179	orf, hypothetical protein; Unknown function; phosphohistidine phosphatase
DNA Recombination & Repair	3/3	*ruvC*	backbone	ED198-E-1	< 0.0001	< 0.0001	Holliday junction nuclease; resolution of structures; Macromolecule synthesis, modification
		xerD	backbone	ED144-E-6	< 0.0001	0.0002	site-specific recombinase; Macromolecule synthesis, modification: DNA- replication, repair, modification
		Z1201	43 O-Island	ED201-H-5	< 0.0001	0.0517	orf; Unknown function, EHEC-specific, UvrD/REP helicase-like protein
Transport protein	3/3	*tolQ*	backbone	ED152-E-9	< 0.0001	< 0.0001	inner membrane protein, membrane-spanning, maintains integrity of cell envelope; tolerance to group A colicins; Colicin-related functions
		tolA	Junction	ED198-C-3	< 0.0001	< 0.0001	membrane spanning protein, required for outer membrane integrity; Colicin-related functions
		betT	backbone	ED201-A-1	< 0.0001	0.0322	high-affinity choline transport; Transport of small molecules: Other
Cell Protection Systems	3/3	*cutC*	backbone	ED203-G-2	< 0.0001	0.0139	copper homeostasis protein; Protection responses: Detoxification
		yhjA	backbone	ED16-F-11	< 0.0001	< 0.0001	putative enzyme; Not classified, a predicted cytochrome c peroxidase
		hdeA	backbone	ED159-G-6	< 0.0001	< 0.0001	orf, hypothetical protein; Unknown function, stress response protein acid-resistance protein;
Hypothetical protein	10/10	*hfq*	backbone	ED185-H-8	< 0.0001	0.0279	host factor I for bacteriophage Q beta replication, a growth-related protein; unknown function
		yhgA	backbone	ED15-D-2	0.0029	< 0.0001	orf, hypothetical protein; Unknown function, transposase
		yheO	backbone	ED51-C-3	< 0.0001	0.5491	orf, hypothetical protein; Unknown function, DNA-binding transcriptional regulator
		ydeK	backbone	ED121-D-5	< 0.0001	0.0099	orf, hypothetical protein; Unknown function, putative lipoprotein/autotransporter; Extended Signal Peptide of Type V secretion system
		yiaF	backbone	ED141-F-8	< 0.0001	0.2029	orf, hypothetical protein; Unknown function, putative outer membrane lipoprotein
		Z1205	43 O-Island	ED207-F-11	< 0.0001	< 0.0001	orf; Unknown function, EHEC-specific.
		Z2973	76 O-Island	ED200-A-11	< 0.0001	< 0.0001	unknown protein encoded by prophage CP-933T, EHEC-specific.
		Z2256/Z2257	46 O-Island/Junction	ED163-E-10	< 0.0001	< 0.0001	Rhs element protein/Rhs element protein, Rhs family protein [Cell envelope biogenesis, outer membrane]
		Z0406/Z0407	backbone	ED1-A-9	< 0.0001	0.0205	hypothetical protein/putative transcription factor, ANK; ankyrin repeats; ankyrin repeats mediate protein-protein interactions
		yagU	backbone	ED82-G-12	< 0.0001	auxotroph	orf; Unknown function
Total	66/91						

References:

1. Hayashi, T., Kato, T., Furukawa, K. Respiratory chain analysis of Zymomonas mobilis mutants producing high levels of ethanol. Appl Environ Microbiol. 2012;78(16):5622-9.

2. Datsenko, KA., Wanner, BL. One-step inactivation of chromosomal genes in Escherichia coli K-12 using PCR products. Proc Natl Acad Sci U S A. 2000;97(12):6640-5.

3. Baba, T., et al. Construction of Escherichia coli K-12 in-frame, single-gene knockout mutants: the Keio collection. Mol Syst Biol. 2006;2:2006 0008.

4. Doublet, B., et al. Antibiotic marker modifications of lambda Red and FLP helper plasmids, pKD46 and pCP20, for inactivation of chromosomal genes using PCR products in multidrug-resistant strains. J Microbiol Methods. 2008;75(2):359-61.

5. Jones, SA., et al. Respiration of Escherichia coli in the mouse intestine. Infection and immunity. 2007;75(10):4891-9.

6. Chen, YM, Lin, EC. Regulation of the adhE gene, which encodes ethanol dehydrogenase in Escherichia coli. J Bacteriol. 1991;173(24):8009-13..

7. Unden, G., et al. Control of FNR Function of Escherichia coli by O2 and Reducing Conditions. J Mol Microbiol Biotechnol. 2002;4(3):263-8.

8. Garriga, X., et al. nrdD and nrdG genes are essential for strict anaerobic growth of Escherichia coli. Biochemical and biophysical research communications. 1996;229(1):189-92.

9. Golby, P., Davies, S., Kelly, DJ., Guest, JR., Andrews, SC. Identification and Characterization of a Two-Component Sensor-Kinase and Response-Regulator System (DcuS-DcuR) Controlling Gene Expression in Response to C4-Dicarboxylates in Escherichia coli. J Bacteriol. 1999 ;181(4):1238-48.

10. Ganesh, I., Ravikumar, S., Lee, SH., Park, SJ., Hong, SH. Engineered fumarate sensing Escherichia coli based on novel chimeric two-component system. Journal of biotechnology. 2013;168(4):560-6.

11. Janausch, IG., Zientz, E., Tran, QH., Kröger, A., Unden, G. C4-dicarboxylate carriers and sensors in bacteria. Biochimica et Biophysica Acta (BBA) - Bioenergetics. 2002:39-56.

12. Brenner, S. The genetics of Caenorhabditis elegans. Genetics. 1974;77(1):71-94.

13. Sato, K., et al. Caenorhabditis elegans SNAP-29 is required for organellar integrity of the endomembrane system and general exocytosis in intestinal epithelial cells. Mol Biol Cell. 2011;22(14):2579-87.

14. Strockbine, NA.,et al. Two toxin-converting phages from Escherichia coli O157:H7 strain 933 encode antigenically distinct toxins with similar biologic activities. Infection and immunity. 1986;53(1):135-40.

15. Yu, SL., Ko, KL., Chen, CS., Chang, YC., Syu, WJ. Characterization of the distal tail fiber

locus and determination of the receptor for phage AR1, which specifically infects Escherichia coli O157:H7. J Bacteriol. 2000;182(21):5962-8.
16. Chou, TC., et al. Enterohaemorrhagic *Escherichia coli* O157:H7 Shiga-like toxin 1 is required for full pathogenicity and activation of the p38 mitogen-activated protein kinase pathway in Caenorhabditis elegans. Cellular microbiology. 2013;15(1):82-97.
17. Valdivia, RH., Falkow, S. Bacterial genetics by flow cytometry: rapid isolation of Salmonella typhimurium acid-inducible promoters by differential fluorescence induction. Molecular microbiology. 1996;22(2):367-78.
18. Muniesa, M., Serra-Moreno, R., Acosta, S., Hernalsteens, JP., Jofre, J. Use of the lambda Red recombinase system to produce recombinant prophages carrying antibiotic resistance genes. BMC Mol Biol. 2006;7.
